# Understanding In-Liquid Sample Environments for Infrared Nanospectroscopy of Soft Materials

**DOI:** 10.1021/acs.analchem.6c03284

**Published:** 2026-08-31

**Authors:** Maria Eleonora Temperini, Cecilia Spedalieri, Antonia Intze, Valeria Giliberti, Michele Ortolani, Janina Kneipp, Alexander Veber

**Affiliations:** † Institute for Electronic Structure Dynamics, Helmholtz-Zentrum Berlin für Materialien und Energie GmbH, Albert-Einstein-Straße 15, 12489 Berlin, Germany; ‡ Department of Chemistry, 9373 Humboldt-Universität zu Berlin, Brook-Taylor-Straße 2, 12489 Berlin, Germany; § Department of Physics, 9311 Sapienza University of Rome, Piazzale Aldo Moro 5, 00185 Rome, Italy

## Abstract

Recent advances in infrared (IR) scattering-type scanning near-field optical microscopy (s-SNOM) have enabled label-free IR spectroscopy of biomolecules and cells in native aqueous environments with nanoscale spatial resolution. This is achieved using an ultrathin membrane that prevents direct mechanical interaction between the atomic force microscope probe and the liquid while permitting the evanescent optical field to detect the underlying sample. In this configuration, the membrane influences the far- and near-field response of the microscope, requiring the multilayered sample system to be considered when interpreting near-field interactions. Here, we present a systematic study of ultrathin silicon nitride (SiN_*x*_) and silicon carbide (SiC) membranes, investigating their near-field optical response both in a dry condition and in contact with aqueous solutions of bovine serum albumin and DNA molecules. In addition, we demonstrate nanoscale IR (nano-IR) imaging and spectroscopy of individual aggregates of α-synuclein protein in the form of fibrils, in a liquid environment under a SiN_*x*_ membrane. Results indicate that the membrane material not only influences the available transparency window but also introduces spectral changes determined by the optical properties of the membranes and the adhesion of the sample to the substrate. Furthermore, we show that finite dipole modeling can reproduce the near-field spectra of membrane-biomolecule stacked samples, providing a consistent framework for interpreting experimental nano-IR results. This comparative study provides insights relevant for selecting membrane materials for mid-IR s-SNOM experiments in liquid, highlighting the advantages and limitations of SiN_*x*_ and SiC materials, while identifying potential sources of artifacts and deviations from the idealized model.

## Graphical abstract

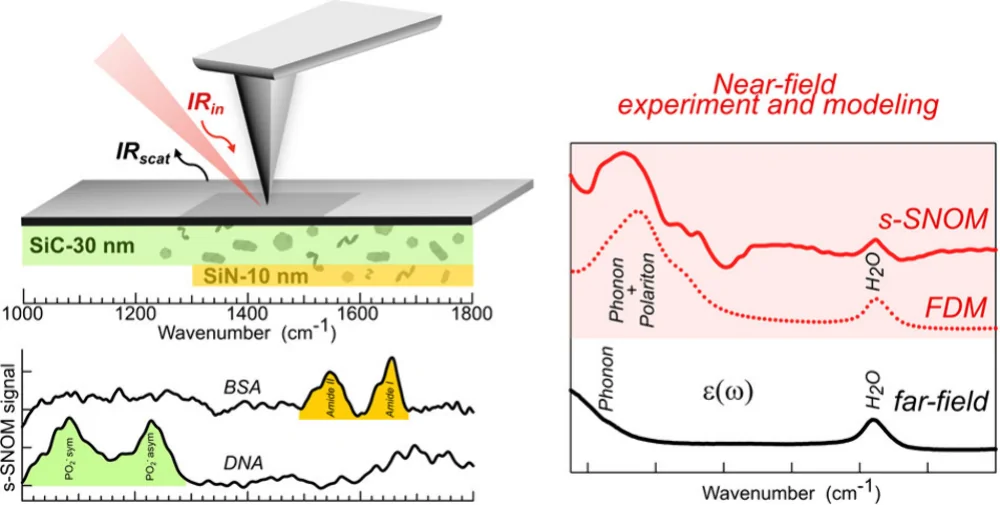

## Introduction

Nanoscale spectroscopy, in the form of scattering-type scanning near-field optical microscopy (s-SNOM), is a powerful technique for investigating the optical properties of materials and biological systems from the visible to the terahertz (THz) range, offering a spatial resolution down to approximately 10 nm.^1,2^ A focused light beam illuminates the sharp tip of an atomic force microscope (AFM) and the backscattered light is detected interferometrically, carrying local optical information about the tip–sample near-field interaction.^3,4^ While most s-SNOM experiments are performed on dry samples, several studies have demonstrated that near-field measurements can also be carried out in liquid environments.^5–12^ This capability is particularly crucial for biological and biochemical applications, as variations in molecular structure, function, and interactions typically occur at the nanometer scale and are often preserved only in aqueous environments.^13,14^ At the same time, the use of liquid environments poses significant challenges for mid-infrared (mid-IR) spectroscopy, due to the strong absorption of water.^15,16^ To overcome this limitation in nanoscale IR spectroscopy, two main approaches have been developed. The first uses total internal reflection (TIR) configurations, typically with silicon (Si), germanium (Ge), or zinc selenide (ZnSe) prisms,^5^ where the liquid sample is placed on the prism surface. In this setup, the IR light undergoes TIR inside the prism, generating an evanescent field that penetrates only a few hundred nanometers into the liquid, allowing local probing without excessive absorption by water. Similar TIR configurations have also been employed in liquid-phase AFM-IR nanospectroscopy, where local IR absorption is detected through photothermal expansion-induced cantilever oscillations.^17^ However, AFM-IR measurements in aqueous environments remain challenging due to water absorption and liquid-induced damping of the cantilever response,^18^ which can reduce the signal-to-noise ratio (SNR). Several approaches have been explored to mitigate these limitations, including the use of deuterated water to shift solvent absorption bands away from the spectral region of interest,^19^ tapping-mode operation to reduce liquid-related background contributions,^20^ and alternative ATR-based illumination schemes that avoid the need for IR-transparent prisms.^21^ In the case of s-SNOM, liquid-induced damping of the cantilever oscillation is also critical, as the mechanical oscillation is used for signal demodulation.^22^ The second approach for in-liquid s-SNOM experiments involves nanometer-thick membranes made of mid-IR transparent materials, such as silicon nitride (SiN_*x*_),^9^ silicon carbide (SiC),^7^ graphene,^10,23^ and metal-oxide,^24^ acting as an interface between the AFM tip and the liquid environment. The membrane-based configuration not only avoids direct IR propagation through the liquid solution, probing only the near-field region of the AFM tip through the membrane and thereby mitigating water absorption, but also it prevents tip contamination and oscillation damping that would otherwise arise from having the AFM tip in the liquid environment,^25^ although at the cost of losing direct AFM topographical information from the sample. At the same time, the electromagnetic response of the membrane material becomes critical for mid-IR s-SNOM in liquid. Both transparency in the relevant spectral range and polaritonic behavior, such as surface phonon polaritons (SPhPs) supported by polar materials, can significantly influence the near-field contrast and the overall SNR as well as introduce spectral distortions.^26–28^

Being capable to detect the amplitude and phase of the near-field optical signal, s-SNOM techniques provide complementary information on the local dielectric permittivity at IR frequencies and, thereby, on the chemical composition of the material at the nanoscale.^29^ Proper interpretation of the observed data requires modeling of the near-field tip–sample interaction.^30,31^ It has been shown that for weak oscillators, the amplitude is primarily sensitive to the real part (Re­[ε]) of the local dielectric function and, therefore, similar to the refractive index and reflectivity, while the phase-shift is more strongly influenced by the imaginary part (Im­[ε]) of the dielectric response, which is directly linked to the extinction coefficient and molecular absorption bands.^32,33^ Obtaining nano-IR spectra of biomaterials through a membrane material only partially fulfills the condition of the weak oscillator regime, since the commonly used SiN_*x*_ and SiC for such experiments are strongly polar dielectrics. Therefore, the data collected in-liquid through the membrane should be analyzed carefully taking into account both the electromagnetic response of the membrane as well as that of the solution and of the object of interest underneath.

In this work, we present a systematic study of SiN_*x*_ (hereafter referred to as SiN) and SiC free-standing membranes, investigating their near-field optical response both in dry conditions and in contact with aqueous solutions, based on experiments and modeled data. Measurements are performed using broadband synchrotron radiation in the 800–2000 cm^–1^ spectral range,^34,35^ enabling nanospectroscopy in the fingerprint region of protein and DNA molecules in aqueous solutions. Moreover, we demonstrate imaging and spectroscopy of supramolecular structures of aggregates of α-synuclein protein in a liquid environment. The s-SNOM measurements are complemented by far-field Fourier-transform infrared (FTIR) experiments. From the far-field spectra, we model the complex dielectric functions of membrane materials and biomolecules, which serve as input parameters for near-field simulation results, based on the finite dipole model (FDM).^36^ This framework enables numerical calculations of the near-field optical amplitude and phase signals, providing a consistent basis for interpreting the experimental nano-IR results. We also discuss the factors that may influence the experimental spectra and their deviation from the modeling. The outcomes of this work have implications for selecting optimal membrane materials for mid-IR s-SNOM in liquid environments. Specifically, we demonstrate the effectiveness of nano-IR s-SNOM measurements using ultrathin membranes for nanoscale vibrational spectroscopy of biological systems, including proteins and nucleic acids, under conditions close to their native state in aqueous solutions.

## Experimental Section

**MEMBRANES** SiN_*x*_ membranes were purchased from Norcada Inc. (Canada). For the s-SNOM experiments, the NX5025Z model was employed, featuring a SiN_*x*_ window (250 × 250 μm^2^, 10 nm thickness) on a Si frame (5 × 5 mm^2^, 200 μm thick). SiC (3C-SiC polytype) membranes were purchased from Silson Ltd. (UK). The model M1002083 was employed for the s-SNOM experiments. The SiC window features nominal dimensions of 150 × 150 μm^2^ and a thickness of 30 nm on a Si frame (5 × 5 mm^2^, 380 μm thick). For the s-SNOM measurements in a liquid environment, both SiN and SiC membranes were pretreated by air plasma for 10 min to enhance wettability. Subsequently, they were mounted on a custom-made aluminum holder with a 2.5 mm through-hole at its center. The membranes were fixed on the top side of the holder by placing its frame into a recess matching its dimensions and securing it with a thin layer of neutral transparent sealant. The liquid solution (∼25 μL) was then introduced through the opening on the back side of the holder, which was subsequently sealed with adhesive tape. The chamber was filled to near-capacity, leaving a minimal air gap to isolate the liquid phase from the underside adhesive tape. A technical drawing of the sample environment assembly is reported in SI, section S-X.

**SAMPLE SOLUTIONS** Protein and nucleic acid solutions were prepared in Milli-Q water, utilizing bovine serum albumin (BSA) at a concentration of 0.55 g/mL (Sigma-Aldrich, Steinheim, Germany) and low molecular weight salmon sperm DNA at 1% (w/w) (Sigma-Aldrich, St. Louis, USA). α-Synuclein wild-type protein was expressed and purified from competent *E. coli* BL21 [DE3] cells (Thermo Fisher Scientific, Waltham, MA), as described previously.^37^ Purified protein aliquots were stored at −80 °C and prepared at a final concentration of 50 μM in aggregation buffer (20 mM potassium phosphate, pH 7.2, 100 mM KCl, 5 mM MgCl_2_, 0.05% NaN_3_). Aggregation assays were performed at 37 °C with constant agitation at 800 rpm for 144 h.

**S-SNOM MEASUREMENTS** Nanospectroscopy measurements were performed using a s-SNOM microscope (neaScope, Attocube, Germany) equipped with a nano-FTIR module. This module was coupled to the broadband synchrotron radiation of the IRIS beamline at the BESSY II storage ring facility (Helmholtz-Zentrum Berlin). The employed AFM cantilever was a Si-bulk model with a Pt–Ir coating (nominal tip radius around 60 nm), featuring a resonance frequency (Ω) of 300 kHz and a spring constant of 37 N/m (ANSCM-PA5, Applied NanoStructures Inc., USA). The AFM probe was operated in tapping mode at a frequency close to its resonance Ω, with tapping amplitude of 115 nm and set-point of 70%. The signal was collected by a liquid nitrogen-cooled mercury cadmium telluride (HgCdTe) detector and demodulated at the second harmonic 2Ω of the cantilever resonance frequency,^38^ which provides an optimal compromise between near-field sensitivity and SNR. The harmonic dependence of the near-field response and the corresponding signal decay were evaluated by retract curve measurements (see SI, Section S-IX). All measurements were conducted in an environment purged with dry nitrogen. Spectra were acquired with a spectral resolution of 6.25 cm^–1^, an integration time of 100 ms per spectral pixel, 10 averages per measurement, and 5 repetitions per sample. Measurements in the dry state, as well as pure water and α-synuclein, were referenced against clean Si. BSA and DNA solution spectra were referenced against pure water. The interferometric data were processed using an in-house-developed script implemented in the open-source SciLab software. Raw interferograms of the 5 repetitions for each sample were first averaged and then apodized with an asymmetric three-term Blackman-Harris window and a zero-filling factor of 4. The apodized interferograms were then Fourier-transformed to obtain frequency-domain spectra, which were referenced to the corresponding and treated reference spectrum. A linear baseline was subsequently subtracted from the optical phase-shift spectra to remove residual offsets. The white-light maps were acquired with the reference mirror of the interferometer fixed at the position corresponding to the maximum of the interferogram. The number of pixels in the *x*–*y* plane was chosen to achieve a spatial resolution of approximately 30 nm, and the integration time per pixel was 20 ms. The resulting maps were processed using the open-source software Gwyddion^39^ to apply plane corrections and adjust the color scale for visualization.

**FAR-FIELD MEASUREMENTS OF SiN AND SiC** For more precise measurements of the SiN and SiC dielectric function, free-standing windows with a thickness of 500 nm were measured in reflection at normal incidence using an FTIR spectrometer (Vertex 70v, Bruker, Germany) - see section S–I of SI. For SiN_*x*_, the NX10300E model (Norcada Inc., Canada) was used, with a 3 × 3 mm^2^ window on a Si frame (10 × 10 mm^2^, 200 μm thick). For SiC, the model M1002304 (Silson Ltd., UK) was used, with a 5 × 5 mm^2^ window on a Si frame (10 × 10 mm^2^, 381 μm thick). Reflection spectra of the SiN and SiC membranes were referenced against gold.

**ATR MEASUREMENTS OF LIQUID SOLUTIONS**Attenuated total reflection (ATR) measurements of water and the same BSA and DNA solutions used for the s-SNOM experiments were performed using an FTIR spectrometer (Vertex 70v, Bruker, Germany) with an internal Globar source, a 4 mm aperture size, and a DTGS detector. The setup utilized a Ge prism with an internal reflection angle of 45°.

**MODELING OF DIELECTRIC FUNCTIONS** The SiN and SiC data measured on 500 nm thick membranes were modeled using the open-source software Focus 2.0,^40^ applying a thin-film reflectance model in which the complex dielectric function was approximated by a sum of Lorentzian oscillators and a Gaussian contribution for SiN and a single Lorentzian oscillator for SiC. ATR spectra of BSA and DNA solutions were referenced against pure water. In the model, the experimental configuration was simplified by treating the samples as thin effective layers in a transmission-like geometry with estimated thicknesses of 50 nm for BSA and 10 nm for DNA. Experimental data were fitted by using a transmittance model based on a sum of Lorentzian oscillators. Detailed model parameters are reported in the SI (Sections S–I and S–III). The dielectric functions of water^41^ and SiO_2_,^42^ required for referencing in near-field simulations, were obtained from literature values available in a refractive index database.^43^

**MODELING OF S-SNOM DATA** Modeling of the s-SNOM spectra was performed using the open-source Python package *snompy*.^44^ The simulated signals were demodulated at the second harmonic using the Q-average method within the FDM framework (see details in the SI, Section S–VI). The AFM probe was modeled as an ellipsoid with a radius *r* = 60 nm and a semimajor axis *L* = 200 nm, and the tapping amplitude was set to 115 nm to replicate experimental conditions. In the model, the sample was represented as a multilayer system arranged along the *z*-axis, consisting of an upper semi-infinite environment (air), a membrane layer (10 nm for SiN or 30 nm for SiC), a molecular layer, and a lower semi-infinite environment of water. The empty cell and the water-filled cell were modeled by replacing the biomolecular layer and water environment with a single semi-infinite environment of air and water, respectively. For measurements referenced to Si, including empty and water-filled membranes as well as α-synuclein fibrils, the dielectric function of the Si substrate was kept constant at 11.7. A 2 nm-thick SiO_2_ layer was included on top of the semi-infinite Si substrate to account for the optical response of the oxide layer. Around 1200–1250 cm^–1^, where the real part of the SiO_2_ dielectric function approaches zero, polaritonic effects enhance the near-field signal of the naturally formed native oxide on Si.^45^ For measurements referenced to water, including BSA and DNA solutions, the samples were modeled as thin molecular layers within the multilayer stack. The effective thicknesses were set to 5 nm for BSA and 30 nm for DNA to reproduce the experimental intensities and were kept constant for the two different membrane materials.

## Results and Discussion

### Near-Field Optical Properties of Free-Standing SiN and SiC Membranes

We first discuss 10 nm-thick SiN and 30 nm-thick SiC free-standing membranes under dry conditions. Figures 1a and 1b report the optical amplitude *s*_2_ and phase-shift φ_2_ signals (colored solid lines) for the SiN and SiC, respectively. In φ_2_ for both materials, we clearly observe resonances located at 985 cm^–1^ and 1130 cm^–1^ for SiN and at 875 cm^–1^ and 965 cm^–1^ for SiC that can be associated with the excitation of SPhPs of the membrane. These features occur within respective Reststrahlen bands of the two membrane materials, in the spectral range where polar dielectrics exhibit a negative Re­[ε] between the transverse (TO) and longitudinal optical (LO) phonon frequencies^46,47^ (see Section S–I of SI for the dielectric functions).

**Figure 1:**
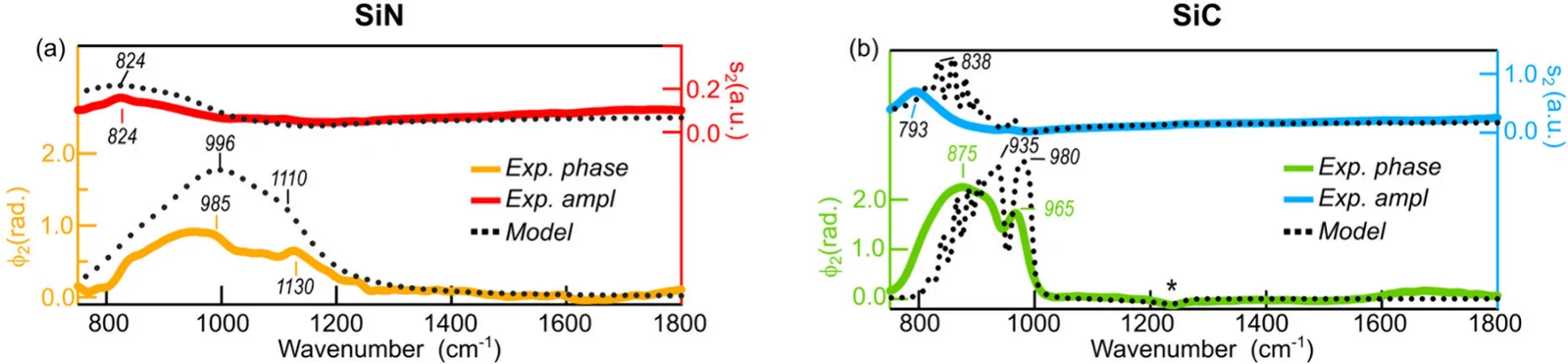
(a) and (b) Experimental (colored solid curves) and modeled (black dotted curves) s-SNOM spectra for membranes in dry state. All data are referenced to Si. (a) φ_2_ (orange, left axis) and *s*_2_ (red, right axis) signals for a SiN membrane in the dry state. (b) φ_2_ (light green, left axis) and *s*_2_ (light blue, right axis) signals for a SiC membrane in the dry state. The negative peak marked with an asterisk originates from the enhanced near-field response of SiO_2_ layer on the Si reference.

The exact frequencies and intensities of the SPhP excitations depend on the geometry of the membrane and the dielectric environment. For nanometer thick films, the reduced thickness can lead to electromagnetic coupling between the SPhPs supported by the two interfaces of the membrane. In a symmetric dielectric environment, such as the air/membrane/air configuration considered here, this coupling can result in the hybridization and splitting of the polaritonic modes into multiple resonances.^28,48^ The presence of the SPhP modes significantly reduces the spectral range accessible for the near-field experiment when compared to the far-field transmission spectra (see Section S–I of SI). In particular, the 10 nm SiN membrane exhibits transparency in the spectral range above 1300 cm^–1^ (Figure 1a), whereas the 30 nm SiC is transparent in the spectral range above 1050 cm^–1^ (Figure 1b), consistent with previous studies of SPhPs in these materials.^49,50^ The FDM near-field spectra of the dry SiN and SiC membranes are shown in the corresponding panels of Figure 1a and 1b (dotted curves). Despite the observed mismatch both in the intensity and the position of the spectral features, likely due to the nonlocal character of the SPhP excitation which is neglected in the dipole model, the FDM calculations allow us to qualitatively describe the near-field experiments. The model predicts well the presence of multiple SPhP resonances. The position of these components for the 10 nm SiN membrane is approximately 996 and 1110 cm^–1^, respectively, and matches well the experimentally observed data (Figure 1a). For the 30 nm SiC membrane, the value of 935 cm^–1^ obtained in the model is significantly shifted with respect to the experimentally observed value (Figure 1b), possibly due to mechanical strain in the membrane material, but at the same time, the predicted position of the mode at approximately 980 cm^–1^ agrees well with the observed value (Figure 1b). Variations in spectral shape and peak position compared to previous near-field studies^51,52,42^ can be attributed to differences in size, geometry, and environment of the SiN and SiC systems.

### Spectra of Biomolecules in Aqueous Environments through Ultrathin Membranes

**Water**To understand the dependency of the spectral response of soft materials measured in the s-SNOM configuration through an ultrathin membrane we first measure spectra of the membranes with water underneath. The corresponding *s*_2_ and φ_2_ spectra are shown in Figures 2a and 2b for SiN and SiC, respectively. The membrane peaks in the phase-shift signal associated with SPhP modes of the membrane material are red-shifted compared to the dry state in both materials. This shift is reproduced by the model results (black dotted curves in Figure 2a and 2b) and originates from the increased dielectric loading introduced by water. The higher dielectric permettivity of water modifies the SPhP resonance condition, shifting it toward more negative values of the real part of the permittivity of the polar material. For SiC and SiN, this condition is reached at lower frequencies within the Reststrahlen band, resulting in the observed red shift. The dielectric-environment dependence of phonon-polariton modes for thin polar dielectric systems has been previously reported.^53,54^ The phase-shift signal clearly reveals the absorption band associated with the OH bending mode of water, with peak positions at 1648 cm^–1^ in the case of the SiN membrane and 1641 cm^–1^ for SiC. The band frequency in the modeled data is consistent with experimental observations for both materials.

**Figure 2:**
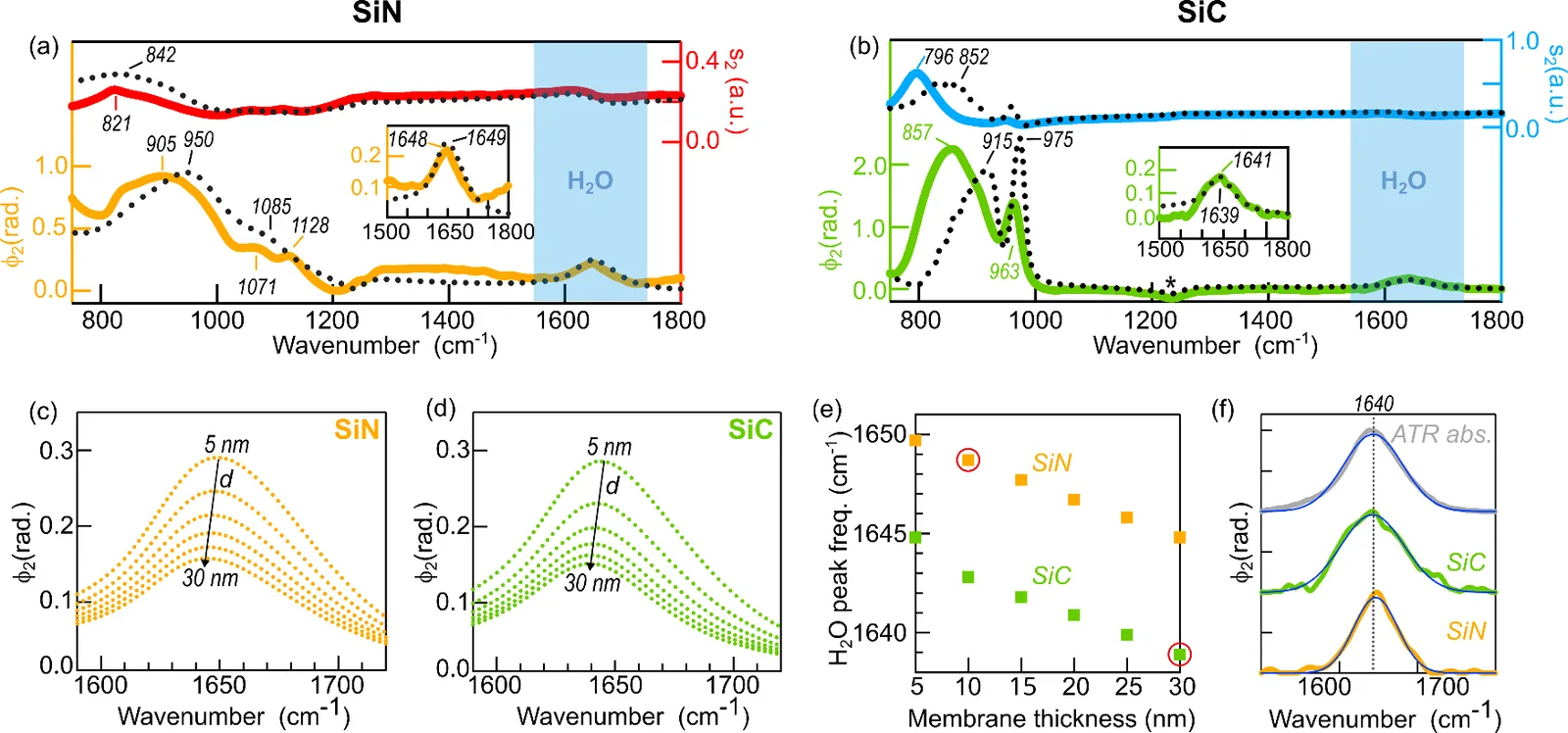
Experimental (colored solid curves) and modeled (dotted curves) s-SNOM spectra for membranes in liquid environment. (a) φ_2_ (orange, left axis) and *s*_2_ (red, right axis) signals for a SiN membrane with water underneath. (b) φ_2_ (light green, left axis) and *s*_2_ (light blue, right axis) signals for a SiC membrane with water underneath. The negative peak marked with an asterisk originates from the enhanced near-field response of SiO_2_ layer on the Si reference. All data are referenced to Si. (c, d) Modeled φ_2_ s-SNOM signal of the OH bending mode under SiN and SiC membranes, respectively, for membrane thicknesses *d* ranging from 5 to 30 nm in 5 nm steps. (e) Peak position of the OH bending mode in the modeled data as a function of membrane thickness for the two materials. Red circles indicate the thickness of SiN and SiC membranes for the experimental measurements in (a) and (b), respectively. (f) Comparison of the OH bending mode peak in the SiN (orange) and SiC (green) s-SNOM measurements to ATR measurements (gray) of pure water. The ATR absorbance intensity is normalized to s-SNOM data. The blue curves are Gaussian fitting curves.

We infer that the difference in peak position of the water signal in the presence of the two membranes reflects variations in the near-field coupling between the tip and the membrane-liquid stack, where both the material composition and the membrane thickness influence the resonance frequency. Figures 2c and 2d show the modeled absorption peak of the OH bending mode of water in the presence of SiN and SiC, respectively, highlighting a red shift of the peak position as the membrane thickness increases. Figure 2e reports the peak position as a function of membrane thickness for both materials, showing that measurements performed with SiC consistently result in lower peak frequencies compared to SiN for the same membrane thickness. The FDM calculations performed for SiN and SiC membranes of varying thickness thus reproduce the experimentally observed trend, supporting the interpretation that the peak shift depends on both membrane thickness and material properties. We also observe that the band is slightly broader in the SiC data (FWHM ∼ 95 cm^–1^) than in the SiN case (FWHM ∼ 66 cm^–1^), as obtained from the Gaussian fits (blue curves in Figure 2f). At the same time, the band shape remains largely symmetric and overall only weakly affected by the membrane environment. Comparison with far-field measurements shows good agreement in both band shape and peak position with ATR spectra acquired using a Ge prism, showing a FWHM ∼ 89 cm^–1^ at a peak maximum of 1640 cm^–1^ (gray curve in Figure 2f). A more detailed analysis of the band shape of the OH deformation mode is provided in Section S–II of the SI.

**Bovine Serum Albumin Solution** Under the same experimental conditions, we measured the spectra of BSA water solution using the SiN and SiC membrane. After referencing each spectrum to the corresponding spectrum of pure water, the amide I (C=O stretching) and amide II (N–H bending coupled to C–N stretching) modes associated with the peptide bonds^55^ are clearly resolved in both amplitude and phase-shift spectra (Figures 3a and 3c). The model reproduces well the phase-shift response of the protein (black dotted curves in Figures 3b and 3d). The experimental positions for amide I and amide II bands are at 1652 cm^–1^ and 1545 cm^–1^ for SiN, corresponding well to the model prediction of 1657 cm^–1^ and 1547 cm^–1^, respectively, and at 1656 cm^–1^ and 1546 cm^–1^ for SiC, in agreement with the predicted positions of 1655 cm^–1^ and 1548 cm^–1^, respectively. Weaker bands are observed in the 1340–1480 cm^–1^ region and include contributions from amide III components and side-chain vibrations. Also, these modes are predicted by the FDM model.

**Figure 3:**
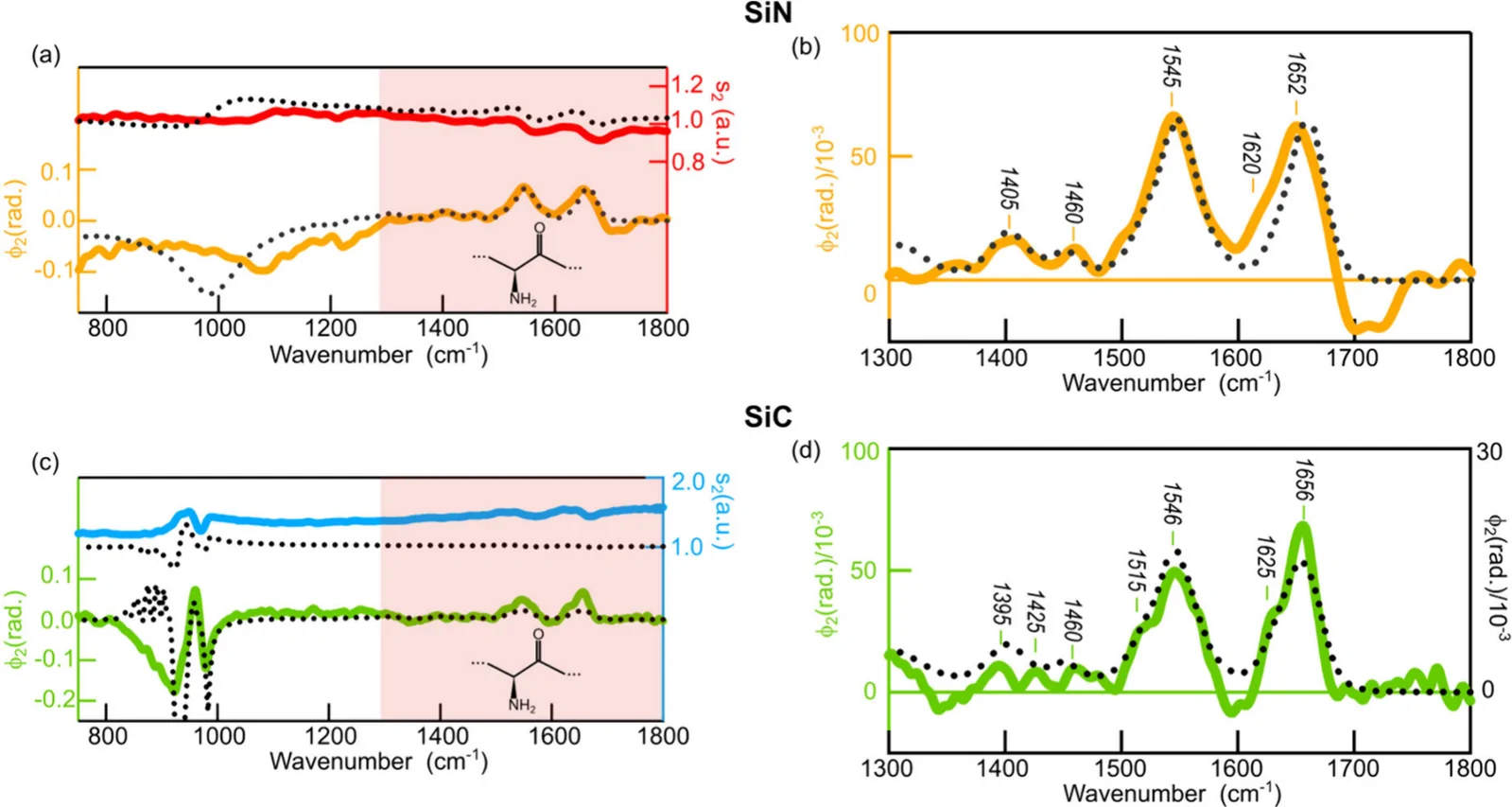
Experimental and modeled s-SNOM spectra of BSA solution. All data are referenced to water measurements. The black dotted lines represent the corresponding modeled results. (a) φ_2_ (orange, left axis) and *s*_2_ (red, right axis) spectra for BSA solution probed through a SiN membrane. (b) Magnification of φ_2_ signal from the reddish area of (a), highlighting the molecular absorption region. (c) φ_2_ (light green, left axis) and *s*_2_ (light blue, right axis) for BSA solution through a SiC membrane. (d) Magnification of φ_2_ signal from the reddish area of (c) (right axis for the light-green experimental curve, left axis for the modeled black curve).

In the case of SiN membranes, both the peak intensities and positions show good agreement between the theoretical and experimental spectra (Figure 3b). In contrast, spectra acquired through the SiC membrane reveal additional peaks compared to the modeled spectra (Figure 3d). The observed extra spectral components could be due to differences in protein–surface interactions that differ for the two membrane materials. IR studies of proteins adsorbed on solid substrates have shown that surface adhesion can induce shifts in band positions and the appearance of additional spectral components associated with changes in molecular orientation or local conformation.^56^ Such effects could also account for the higher intensity of the amide bands observed experimentally with the SiC membrane compared to the respective modeled results, noting that the spectra in Figure 3d are plotted on different scales. For both SiN and SiC, the φ_2_ signal becomes negative at frequencies approaching the SPhP bands of the respective membrane material (Figures 3a and 3c). This behavior likely originates from the strong spectral response of the membrane in this region discussed above (Figures 1a and 1b). Because the near-field signal depends on the complex optical contrast within the tip–sample interaction volume, even small differences in refractive index between pure water and the BSA solution can modify the effective near-field interaction. As a result, membrane-related background features are not perfectly compensated when the spectra are referenced to water. The transition between positive and negative phase-shift is observed at approximately 1300 cm^–1^ for the 10 nm SiN membrane (Figure 3a) and 1050 cm^–1^ for the 30 nm SiC membrane (Figure 3c).

**DNA Solution** The spectra of a DNA water solution obtained using the SiN membrane, referenced to the corresponding water measurement, are reported in Figures 4a and 4b. The overlap between the main DNA vibrational modes and the SPhP resonance of SiN hampers the clear resolution of the absorption bands, impairing a reliable analysis in the 1000–1300 cm^–1^ spectral range. Nevertheless, weaker spectral features and spectral weight can still be observed in φ_2_ around 1350–1500 cm^–1^ and 1600–1750 cm^–1^, assigned to base-sugar and base-pairing vibrational modes, respectively.^57^ These spectral contributions are consistent with the DNA vibrational response observed in the ATR measurements, although differences in relative intensity and the absence of a direct correspondence for all features are observed (see SI, Section S–IV). Figures 4c and 4d display the DNA spectra acquired using the SiC membrane. In this case, two major vibrational bands are clearly identified at 1083 cm^–1^ and 1230 cm^–1^ assigned to PO_2_^–^ symmetric and asymmetric stretching, respectively.^57^ They are accompanied by several weaker features at 1034, 1060, 1120, 1142, 1178, 1210, 1243, and 1264 cm^–1^, marked in Figure 4d.^57^

**Figure 4:**
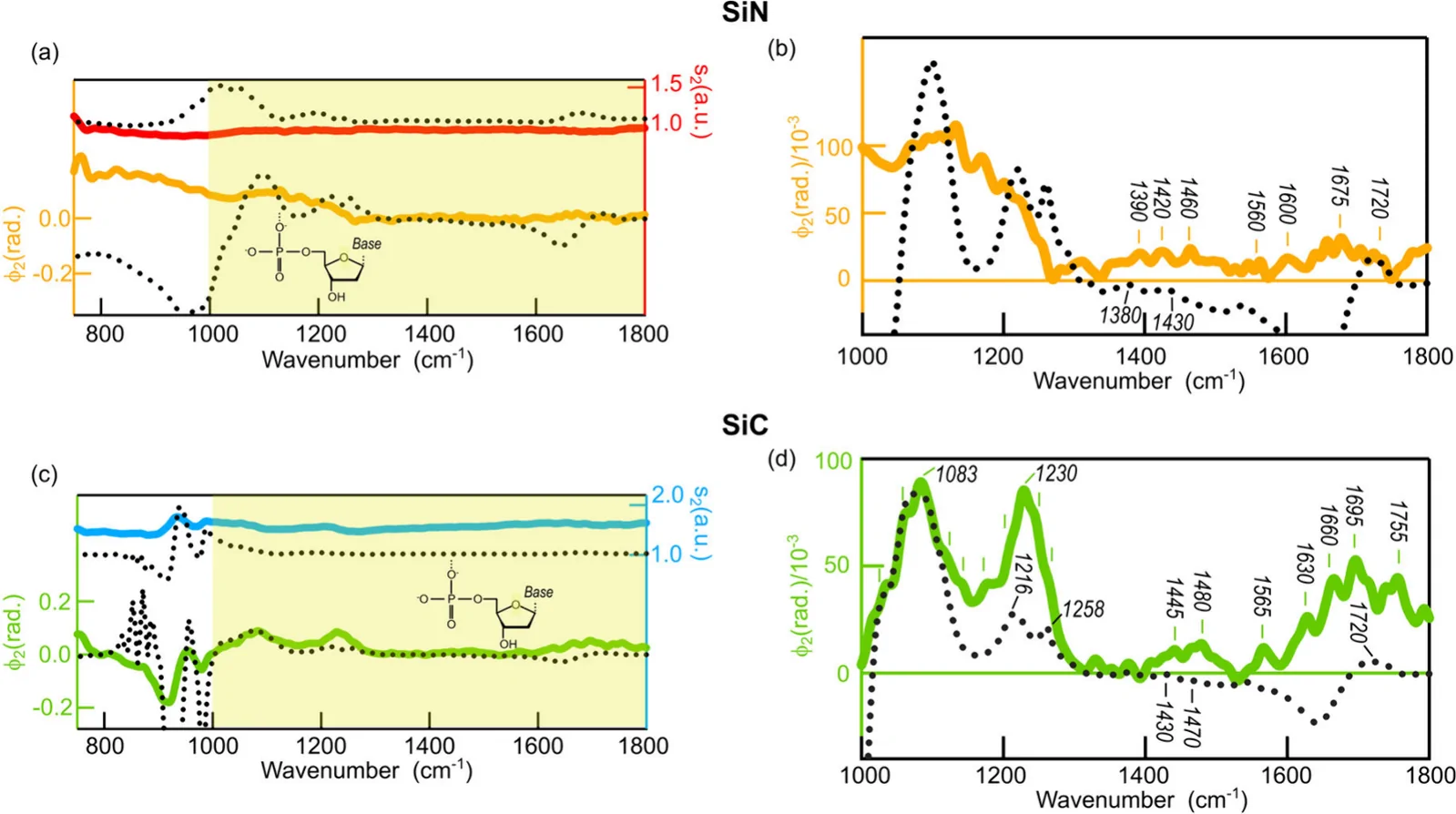
Experimental and modeled s-SNOM spectra of a DNA solution. All data are referenced to water measurements. The black dotted lines represent the corresponding modeled results. (a) φ_2_ (orange, left axis) and *s*_2_ (red, right axis) spectra for a DNA solution probed through a SiN membrane. (b) Magnification of φ_2_ signal from the yellowish area of (a), highlighting the molecular absorption region. (c) φ_2_ (light green, left axis) and *s*_2_ (light blue, right axis) for a DNA solution through a SiC membrane. (d) Magnification of φ_2_ signal from the yellowish area of (c).

Several of these features are also observed in the far-field ATR response of DNA (see SI, section S–IV), supporting their assignment to DNA vibrational modes. The modeled spectrum reproduces the main vibrational bands, although slight frequency shifts and differences in the relative intensity are observed (Figure 4d). These differences likely arise from orientation effects of the DNA strands with respect to the predominantly out-of-plane polarization of the IR near-field at the tip apex, which can depend on both the membrane and DNA film thicknesses and the dielectric function of the surrounding materials.^58^ Such effects are not explicitly accounted for in the model, which assumes an isotropic thin film response. An additional broad contribution between 1650 and 1780 cm^–1^ is clearly found in the experimental data and also qualitatively reproduced by the modeled spectrum, together with weaker features in the 1350–1500 cm^–1^ region. The dip near 1650 cm^–1^ observed in the modeled spectra for both SiN and SiC may originate from an imperfect compensation of water contribution. In fact, the probed water volume in the reference spectrum is larger than that in the DNA spectrum, where part of the probed region is occupied by the DNA layer. A similar effect may also affect the modeled BSA spectra, although it is not clearly visible, because the OH deformation band of water spectrally overlaps with the amide I band. As detailed in the Experimental Section, the model describes the system as a stack of layers (membrane/molecule/water), which represents a simplified geometry compared with the actual configuration of molecules dispersed in solution. In this framework, the thicknesses of 5 and 30 nm of the homogeneous layer for BSA and DNA, respectively, are used as effective parameters to reproduce the experimental signal intensity of the BSA amide I under SiN and DNA PO_2_^–^ stretching mode under SiC. These values are not intended to provide a quantitative estimation of the physical molecule amount, as they depend on the modeled permittivity of BSA and DNA, which is based on ATR measurements (see SI, Section S–III). In particular, under- (or over-) estimation of the molecular layer thickness while modeling the dielectric permittivity can lead to a corresponding over- (or under-) estimation of the oscillator strengths. This correspondingly leads to different thicknesses of molecules inferred from the s-SNOM measurements.

Nevertheless, the molecular absorption bands are clearly resolved in the experimental spectra. Therefore, by considering the near-field interaction volume estimated from retract curve measurements^59^ (see SI, Section S-IX) together with the membrane thickness and solution concentration, we can infer a detection sensitivity on the order of 1 zmol for BSA molecules and 10^–2^ zmol for DNA molecules (see SI, Section S–V).

### Imaging and Spectroscopy of α-Synuclein Fibrils in Liquid

Our results show that SiN membranes are well-suited for the investigation of proteins, providing a sufficient spectral window to observe those vibrational modes that are relevant to characterize protein primary and secondary structure. To explore the capabilities of our approach for probing supramolecular structures of proteins, we investigated α-synuclein, a protein associated with neurodegenerative diseases that can form fibrillar aggregates under physiological conditions.^60^ In Figure 5a, we report an AFM topography of α-synuclein proteins in the mature fibril state deposited on a gold surface, providing initial information on the sample morphology. In Figure 5b, we show a white-light image (i.e., integrated over the total mid-IR bandwidth) of the same kind of sample in the water environment. From the contrast of the optical amplitude signal *s*_2_, we could identify regions with protein aggregates in direct contact with the SiN surface, corresponding to areas of higher signal intensity. To verify the different chemical composition and/or amount of protein in these different regions, we acquired s-SNOM spectra on both the dark and bright areas. In both cases, using clean Si as the reference for the spectra, we identified spectral features that we assigned to the characteristic amide bands at 1565 cm^–1^ (amide II) and 1650 cm^–1^ (amide I), noting that the amide I peak significantly overlaps with the OH bending mode of water as expected (Figure 5c). The different water content in the probed volume is likely responsible for the different intensity ratios of the bands between the dark and bright areas. This interpretation is further supported by the normalized spectral comparison reported in the SI (Section S–VII), where the relative line shape and contribution of the different spectral components are compared. In the yellow curve (corresponding to spot 1 in Figure 5b), we are probably probing protein aggregates directly below the SiN membrane, where the signal is still affected by a contribution from the surrounding water. In the red curve (corresponding to spot 2 in Figure 5b), the water absorption is dominant, but the persistent presence of the amide II band suggests that proteins are also present in the probed volume.

**Figure 5:**
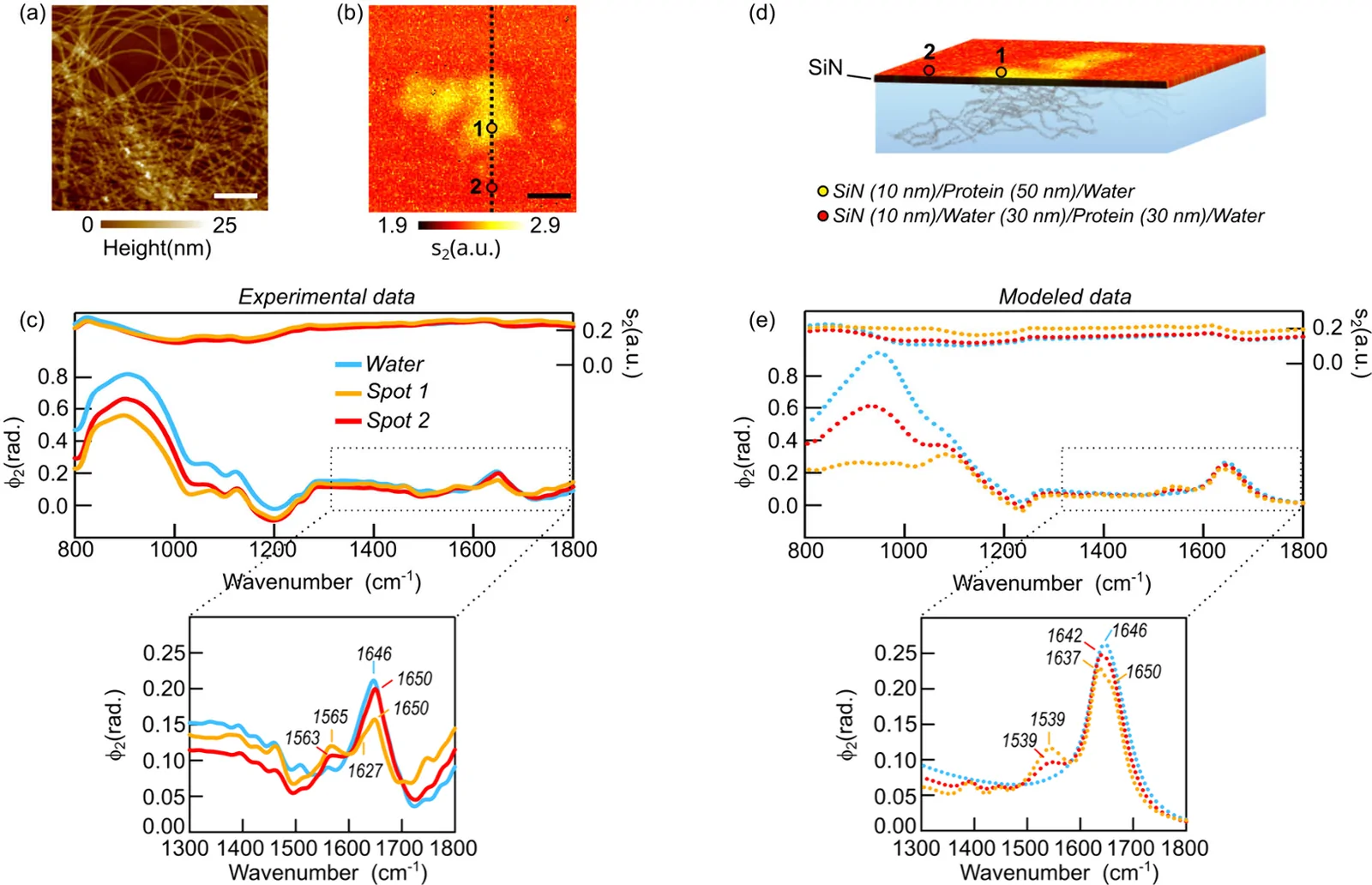
(a) Representative AFM topography of α-synuclein fibrils deposited on a gold surface. Scale bar is 1 μm. (b) s-SNOM optical amplitude *s*_2_ map of α-synuclein fibrils in water solution, acquired through a SiN membrane. Scale bar is 1 μm. (c) φ_2_ (left axis) and *s*_2_ (right axis) signals recorded at the locations marked by spot 1 and 2 in (b), normalized to a Si reference. The light blue curve represents the pure water reference signal. (d) Schematic illustration of a possible fibril arrangement below the SiN membrane, consistent with the observed experimental contrast. The section represents the plane perpendicular to the dotted line indicated in (b). The values of the layer thickness for the modeling of the s-SNOM response in the two positions are also reported. (e) φ_2_ (left axis) and *s*_2_ (right axis) signal results of the model for the two different configurations in (d) under the SiN membrane (yellow and red curves) and for only water (light blue curve).

One possible explanation is that the protein aggregates are at a greater depth relative to the SiN membrane at the spot 2, i.e. possibly part of a fibril cluster not directly attached to the surface, as illustrated in the sketch of Figure 5d. The different content of probed water in the various areas precludes the direct use of the pure water spectrum as the reference for the protein sample spectra. A normalization of the water spectrum would be required to compensate for the varying intensity of the water signal detected across the protein sample regions, but it would still not account for the different dielectric geometry in the different locations. We used the model to propose a possible configuration of the measured areas and report the calculated spectra in Figure 5e. The signal from the bright spot 1 was modeled as an α-synuclein fibril film of 50 nm, while the dark area of spot 2 was modeled as a 30 nm protein film overlaid by a 30 nm layer of water. An alternative configuration for spot 2, consisting of a thinner 10 nm protein layer located directly beneath the membrane, provides a comparable spectral response as reported in the SI section S–VII. Although the model results cannot uniquely determine the actual protein distribution and do not perfectly match the experimental data quantitatively, they support the overall qualitative description of the detected experimental signal, highlighting that the measured spectral response reflects the relative contribution of protein and water.

## Insights and Perspectives

Our findings highlight the critical role of membrane optimization for s-SNOM in liquid environment. Despite the decay of the near-field interaction with distance from the tip,^3,61,62^ the thicker 30 nm SiC membrane still provides a clear and well-resolved near-field response, demonstrating the subsurface sensitivity of the s-SNOM technique.^63^

While the theoretical models successfully capture the main resonance features, some quantitative differences between the experimental data and simulations remain. A spectral mismatch, more evident for SiC, in terms of peak positions of SPhP bands, persists between experiment and simulation for both membranes. Possible origins include uncertainties in the dielectric function of ultrathin SiC and SiN layers, based on deviations from the reflection model used and Lorentzian parameters extracted from the far-field fits (see SI, Section S–I). Additional modeling limitations for the near-field response, such as neglecting the finite lateral size of the membranes, may also influence the overall simulated results. Moreover, in the FDM, the AFM probe is described by an ellipsoid with an effective apex radius and an effective length of the major axis. The apex radius can be roughly inferred from manufacturer specifications or calibration measurements, although it may vary because of tip wearing during the experiment. On the other hand, the effective ellipsoid length, which approximates the elongated geometry of the AFM tip, is chosen within a realistic range based on the tip radius and adjusted to reproduce the experimental data. Variations in these geometrical parameters can affect both the magnitude of the near-field signal and the spectral response,^36,64^ as illustrated in section S–VIII of the SI, where simulations for different ellipsoid lengths are reported.

The observed differences between the experimental results and the model for molecular solutions can be attributed to the inherent complexity of the solid–liquid interface. The FDM assumes a homogeneous and static thin molecular layer, which differs from the actual situation of a dynamic liquid solution where proteins and DNA are distributed nonuniformly, potentially forming clusters, adopting specific orientations, and adhering in different ways to the membrane surface. The near-field response is highly sensitive to molecular orientation, which can affect absorption intensities differently from far-field techniques such as ATR spectra, from which the complex dielectric functions of the materials are extracted for use in the near-field model. For example, polarization and molecular orientation effects can significantly influence the relative peak intensities within the amide I band (corresponding to different secondary structure elements), as the coupling between the vibrational transition dipole moments and the predominantly out-of-plane near-field polarization depends on the orientation of the peptide bonds.^65^ For α-synuclein fibrils, this may explain the lower intensity of the band around 1630 cm^–1^ (β-sheet component) compared to the one at 1650 cm^–1^ (α-helix component) observed in the experimental spectra relative to the modeled data. The β-sheet dipole moments are highly anisotropic and structurally constrained within the fibrils. If they are not favorably aligned with the predominantly tip-oriented near-field, their contribution can be selectively reduced, leading to a lower apparent β-sheet intensity in the measurements.^66,67^ Furthermore, the presence of hydration layers or partially adsorbed biomolecules on the membrane surface can locally alter the dielectric environment, modifying both the amplitude and phase of the tip–sample near-field interaction.

Another important aspect is the role of SPhPs, particularly at the membrane edges. SPhPs may be engineered to enhance the near-field signal, but their behavior in a lossy aqueous environment is difficult to predict with high precision.^54^ Further studies are needed to clarify the interplay between these polaritonic modes and molecular vibrations in liquids. Understanding how the bare-membrane SPhP excitations are modified by the aqueous medium will be essential for future strategies to enhance the sensitivity and frequency range of IR near-field techniques.

## Conclusions

We have demonstrated the effectiveness of s-SNOM for label-free IR spectroscopy of soft matter in native-like liquid environments. By exploiting the high brightness and broadband nature of synchrotron radiation we successfully resolved the vibrational signatures of BSA proteins, DNA molecules, and α-synuclein fibrils in solution in the entire mid-IR range from 800 to 2000 cm^–1^ (wavelengths from 12.5 to 5 μm). Our study showed that both SiN and SiC membranes provide reliable near-field detection, while the SiC membrane offers a broader accessible spectral window that enables DNA characterization. Beyond globular proteins and nucleic acids, the platform enabled the identification of α-synuclein fibrils, demonstrating its capability to probe finely structured protein systems in liquid. The experimental data showed good qualitative agreement with theoretical simulations based on the finite dipole model, effectively capturing the observed absorption bands and the overall trend of the peak shifts when changing the experimental conditions. However, minor quantitative discrepancies remain, likely due to intrinsic limitations of the analytical model,^30,31^ together with not fully accounted aspects of the complex experimental configuration, such as limited lateral size of the membrane windows, molecular orientation, and local clustering. Considering the volume probed in the s-SNOM measurements, we established that our platform could achieve exceptional detection sensitivities at the zeptomole scale, specifically of the order of 1 zmol for BSA and 10^–2^ zmol for DNA. These results confirm that the s-SNOM is a robust and highly sensitive tool for investigating biological and soft material samples even in aqueous environments. This approach opens new frontiers for the monitoring of biological processes, such as protein dynamics and molecular interactions, in their natural state with unprecedented spatial resolution.

## Supplementary Material



## Notes

Additional details on permittivity and FDM modeling, fitting results of OH bending mode peak and calculation of probed molecule amount (PDF)

## References

[1] Chen X., Hu D., Mescall R., You G., Basov D. N., Dai Q., Liu M. (2019). Modern Scattering-Type Scanning Near-Field Optical Microscopy for
Advanced Material Research. Adv. Mater..

[2] Hillenbrand R., Abate Y., Liu M., Chen X., Basov D. N. (2025). Visible-to-THz
near-Field Nanoscopy. Nat. Rev. Mater..

[3] Keilmann F., Hillenbrand R. (2004). Near-Field Microscopy by Elastic Light Scattering from
a Tip. Philos. Trans. R. Soc. London. Ser. A
Math. Phys. Eng. Sci..

[4] Huth F., Govyadinov A., Amarie S., Nuansing W., Keilmann F., Hillenbrand R. (2012). Nano-FTIR
Absorption Spectroscopy of Molecular Fingerprints
at 20 Nm Spatial Resolution. Nano Lett..

[5] O’Callahan B. T., Park K. D., Novikova I. V., Jian T., Chen C. L., Muller E. A., El-Khoury P. Z., Raschke M. B., Lea A. S. (2020). In Liquid
Infrared Scattering Scanning Near-Field Optical Microscopy for Chemical
and Biological Nanoimaging. Nano Lett..

[6] Khatib O., Wood J. D., McLeod A. S., Goldflam M. D., Wagner M., Damhorst G. L., Koepke J. C., Doidge G. P., Rangarajan A., Bashir R., Pop E., Lyding J. W., Thiemens M. H., Keilmann F., Basov D. N. (2015). Graphene-Based Platform for Infrared
Near-Field Nanospectroscopy of Water and Biological Materials in an
Aqueous Environment. ACS Nano.

[7] Veber A., Spedalieri C., Kneipp J. (2025). Nano-Infrared Imaging and Spectroscopy
of Animal Cells in Liquid Environment. Small.

[8] Baù E., Gölz T., Benoit M., Tittl A., Keilmann F. (2024). Nanoscale
Mechanical Manipulation of Ultrathin SiN Membranes Enabling Infrared
Near-Field Microscopy of Liquid-Immersed Samples. Small.

[9] Kaltenecker K. J., Gölz T., Bau E., Keilmann F. (2021). Infrared-Spectroscopic,
Dynamic near-Field Microscopy of Living Cells and Nanoparticles in
Water. Sci. Rep..

[10] Lu Y. H., Larson J. M., Baskin A., Zhao X., Ashby P. D., Prendergast D., Bechtel H. A., Kostecki R., Salmeron M. (2019). Infrared Nanospectroscopy
at the Graphene-Electrolyte Interface. Nano
Lett..

[11] Heberle J., Pfitzner E. (2020). Infrared Scattering-Type Scanning
near-Field Optical
Microscopy of Biomembranes in Water. J. Phys.
Chem. Lett..

[12] Gölz T., Baù E., Zhang J., Kaltenecker K., Trauner D., Maier S. A., Keilmann F., Lohmüller T., Tittl A. (2025). Transient Infrared Nanoscopy Resolves the Millisecond Photoswitching
Dynamics of Single Lipid Vesicles in Water. Nat. Commun..

[13] Ball P. (2008). Water as an
Active Constituent in Cell Biology. Chem. Rev..

[14] Bellissent-Funel M. C., Hassanali A., Havenith M., Henchman R., Pohl P., Sterpone F., Van Der Spoel D., Xu Y., Garcia A. E. (2016). Water Determines
the Structure and Dynamics of Proteins. Chem.
Rev..

[15] Alcaráz M. R., Schwaighofer A., Kristament C., Ramer G., Brandstetter M., Goicoechea H., Lendl B. (2015). External-Cavity Quantum Cascade Laser
Spectroscopy for Mid-IR Transmission Measurements of Proteins in Aqueous
Solution. Anal. Chem..

[16] Fomina P. S., Proskurnin M. A., Mizaikoff B., Volkov D. S. (2023). Infrared Spectroscopy
in Aqueous Solutions: Capabilities and Challenges. Crit. Rev. Anal. Chem..

[17] Dazzi, A. ; Glotin, F. ; Carminati, R. Theory of Infrared Nanospectroscopy by Photothermal Induced Resonance. J. Appl. Phys. 2010, 107 (12). 10.1063/1.3429214.

[18] Jin M., Lu F., Belkin M. A. (2017). High-Sensitivity
Infrared Vibrational Nanospectroscopy
in Water. Light Sci. Appl..

[19] Ramer G., Ruggeri F. S., Levin A., Knowles T. P. J., Centrone A. (2018). Determination
of Polypeptide Conformation with Nanoscale Resolution in Water. ACS Nano.

[20] Mathurin, J. ; Deniset-Besseau, A. ; Bazin, D. ; Dartois, E. ; Wagner, M. ; Dazzi, A. Photothermal AFM-IR Spectroscopy and Imaging: Status, Challenges, and Trends. J. Appl. Phys. 2022, 131 (1). 10.1063/5.0063902.

[21] Yilmaz U., Sam S., Lendl B., Ramer G. (2024). Bottom-Illuminated
Photothermal Nanoscale
Chemical Imaging with a Flat Silicon ATR in Air and Liquid. Anal. Chem..

[22] Hillenbrand R., Knoll B., Keilmann F. (2001). Pure Optical
Contrast in Scattering-Type
Scanning near-Field Microscopy. J. Microsc..

[23] Meireles L. M., Barcelos I. D., Ferrari G. A., de A Neves P. A. A., Freitas R. O., Lacerda R. G. (2019). Synchrotron Infrared Nanospectroscopy
on a Graphene Chip. Lab Chip.

[24] Lu Y. H., Morales C., Zhao X., Van Spronsen M. A., Baskin A., Prendergast D., Yang P., Bechtel H. A., Barnard E. S., Ogletree D. F., Altoe V., Soriano L., Schwartzberg A. M., Salmeron M. (2020). Ultrathin Free-Standing Oxide Membranes
for Electron and Photon Spectroscopy Studies of Solid-Gas and Solid-Liquid
Interfaces. Nano Lett..

[25] Martin, M. J. ; Fathy, H. K. ; Houston, B. H. Dynamic Simulation of Atomic Force Microscope Cantilevers Oscillating in Liquid. J. Appl. Phys. 2008, 104 (4). 10.1063/1.2970154.

[26] Huber A. J., Ocelic N., Hillenbrand R. (2008). Local Excitation
and Interference
of Surface Phonon Polaritons Studied by Near-Field Infrared Microscopy. J. Microsc..

[27] McArdle P., Lahneman D. J., Biswas A., Keilmann F., Qazilbash M. M. (2020). Near-Field
Infrared Nanospectroscopy of Surface Phonon-Polariton Resonances. Phys. Rev. Res..

[28] Mancini A., Nan L., Wendisch F. J., Berté R., Ren H., Cortés E., Maier S. A. (2022). Near-Field Retrieval of the Surface Phonon Polariton
Dispersion in Free-Standing Silicon Carbide Thin Films. ACS Photonics.

[29] Schnell M., Garcia-Etxarri A., Huber A. J., Crozier K. B., Borisov A., Aizpurua J., Hillenbrand R. (2010). Amplitude- and Phase-Resolved near-Field
Mapping of Infrared Antenna Modes by Transmission-Mode Scattering-Type
near-Field Microscopy. J. Phys. Chem. C.

[30] Cvitkovic A., Ocelic N., Hillenbrand R. (2007). Analytical Model for Quantitative
Prediction of Material Contrasts in Scattering-Type near-Field Optical
Microscopy. Opt. Express.

[31] Cigeroglu T., Zeng J., Tran P., O’Callahan B. T., Muller E. A. (2025). Quantitative Model for Infrared Nano-Spectroscopy
in
Liquid. Opt. Express.

[32] Taubner T., Hillenbrand R., Keilmann F. (2004). Nanoscale Polymer Recognition by
Spectral Signature in Scattering Infrared Near-Field Microscopy. Appl. Phys. Lett..

[33] Pollard B., Muller E. A., Hinrichs K., Raschke M. B. (2014). Vibrational Nano-Spectroscopic
Imaging Correlating Structure with Intermolecular Coupling and Dynamics. Nat. Commun..

[34] Peatman W. B., Schade U. (2001). A Brilliant Infrared Light Source at BESSY. Rev. Sci. Instrum..

[35] Veber A., Puskar L., Kneipp J., Schade U. (2024). Infrared Spectroscopy
across Scales in Length and Time at BESSY II. J. Synchrotron Radiat..

[36] Vincent, T. ; Liu, X. ; Johnson, D. ; Mester, L. ; Huang, N. ; Kazakova, O. ; Hillenbrand, R. ; Boland, J. L. Snompy: A Package for Modelling Scattering-Type Scanning near-Field Optical Microscopy. arXiv 2024. 10.48550/arXiv.2405.20948.

[37] Rupert J., Monti M., Zacco E., Tartaglia G. G. (2023). RNA Sequestration
Driven by Amyloid Formation: The Alpha Synuclein Case. Nucleic Acids Res..

[38] Hillenbrand R., Keilmann F. (2000). Complex Optical Constants
on a Subwavelength Scale. Phys. Rev. Lett..

[39] Nečas D., Klapetek P. (2012). Gwyddion: An Open-Source Software for SPM Data Analysis. Cent. Eur. J. Phys..

[40] *Focus 2.0* . https://www.cemhti.cnrs-orleans.fr/pot/software/focus.html.

[41] Hale G. M., Querry M. R. (1973). Optical Constants of Water in the 200-Nm to 200-Μm
Wavelength Region. Appl. Opt..

[42] Franta D., Nečas D., Ohlídal I., Giglia A. (2016). Optical Characterization
of SiO_2_ Thin Films Using Universal Dispersion Model over
Wide Spectral Range. Opt. Micro- Nanometrology
VI.

[43] *Refractive index database* . https://refractiveindex.info/.

[44] Vincent, T. ; Liu, X. ; Johnson, D. ; Mester, L. ; Huang, N. ; Kazakova, O. ; Hillenbrand, R. ; Boland, J. L. Snompy: A Package for Modelling Scattering-Type Scanning Near-Field Optical Microscopy. arXiv 10.48550/arXiv.2405.20948

[45] Shaykhutdinov T., Furchner A., Rappich J., Hinrichs K. (2017). Mid-Infrared Nanospectroscopy
of Berreman Mode and Epsilon-near-Zero Local Field Confinement in
Thin Films. Opt. Mater. Express.

[46] Passler N. C., Gubbin C. R., Folland T. G., Razdolski I., Katzer D. S., Storm D. F., Wolf M., De Liberato S., Caldwell J. D., Paarmann A. (2018). Strong Coupling of
Epsilon-Near-Zero
Phonon Polaritons in Polar Dielectric Heterostructures. Nano Lett..

[47] Passler N. C., Paarmann A. (2017). Generalized 4 ×
4 Matrix Formalism for Light Propagation
in Anisotropic Stratified Media: Study of Surface Phonon Polaritons
in Polar Dielectric Heterostructures. J. Opt.
Soc. Am. B.

[48] Xu, R. ; Crassee, I. ; Bechtel, H. A. ; Zhou, Y. ; Bercher, A. ; Korosec, L. ; Rischau, C. W. ; Teyssier, J. ; Crust, K. J. ; Lee, Y. ; Gilbert Corder, S. N. ; Li, J. ; Dionne, J. A. ; Hwang, H. Y. ; Kuzmenko, A. B. ; Liu, Y. Highly Confined Epsilon-near-Zero and Surface Phonon Polaritons in SrTiO_3_ Membranes. Nat. Commun. **2024**, 2024 15 (1). 10.1038/s41467-024-47917-x. PMC11150425 38834672

[49] Wu Y., Ordonez-Miranda J., Gluchko S., Anufriev R., de Sousa
Meneses D., Del Campo L., Volz S., Nomura M. (2020). Enhanced Thermal
Conduction by Surface Phonon-Polaritons. Sci.
Adv..

[50] Wu K. T., El-Helou Y., Usureau E., Vuillermet E., Kazan M., Lazar M., Gautier G., Woon W. Y., Bruyant A. (2025). Infrared Photoinduced Force Near-Field Spectroscopy
of Silicon Carbide. Appl. Surf. Sci..

[51] Hillenbrand R., Taubner T., Keilmann F. (2002). Phonon-Enhanced
Light – Matter
Interaction at the Nanometre Scale. Nature.

[52] Stiegler J. M., Abate Y., Cvitkovic A., Romanyuk Y. E., Huber A. J., Leone S. R., Hillenbrand R. (2011). Nanoscale Infrared Absorption Spectroscopy
of Individual Nanoparticles Enabled by Scattering-Type near-Field
Microscopy. ACS Nano.

[53] Kim K. S., Trajanoski D., Ho K., Gilburd L., Maiti A., Van Der Velden L., De Beer S., Walker G. C. (2017). The Effect of Adjacent
Materials on the Propagation of Phonon Polaritons in Hexagonal Boron
Nitride. J. Phys. Chem. Lett..

[54] Virmani D., Bylinkin A., Dolado I., Janzen E., Edgar J. H., Hillenbrand R. (2021). Amplitude- And Phase-Resolved Infrared Nanoimaging
and Nanospectroscopy of Polaritons in a Liquid Environment. Nano Lett..

[55] Barth A., Zscherp C. (2002). What Vibrations Tell
Us about Proteins. Q. Rev. Biophys..

[56] Lenk T. J., Ratner B. D., Gendreau R. M., Chittur K. K. (1989). IR Spectral Changes
of Bovine Serum Albumin upon Surface Adsorption. J. Biomed. Mater. Res..

[57] Banyay M., Sarkar M., Gräslund A. (2003). A Library
of IR Bands of Nucleic
Acids in Solution. Biophys. Chem..

[58] Pascual
Robledo I., Maciel-Escudero C., Schnell M., Mester L., Aizpurua J., Hillenbrand R. (2025). Theoretical Description of Infrared
Near-Field Spectroscopy of In- and Out-of-Plane Molecular Vibrations
in Thin Layers. ACS Photonics.

[59] Mooshammer F., Huber M. A., Sandner F., Plankl M., Zizlsperger M., Huber R. (2020). Quantifying Nanoscale Electromagnetic Fields in Near-Field Microscopy
by Fourier Demodulation Analysis. ACS Photonics.

[60] Spillantini M. G., Crowther R. A., Jakes R., Hasegawa M., Goedert M. (1998). α-Synuclein
in Filamentous Inclusions of Lewy Bodies from Parkinson’s Disease
and Dementia with Lewy Bodies. Proc. Natl. Acad.
Sci. U. S. A..

[61] Raschke M. B., Lienau C. (2003). Apertureless Near-Field Optical Microscopy: Tip-Sample
Coupling in Elastic Light Scattering. Appl.
Phys. Lett..

[62] Taubner T., Keilmann F., Hillenbrand R. (2005). Effect of Tip Modulation on Image
Contrast in Scattering-Type near-Field Optical Microscopy. J. Korean Phys. Soc..

[63] Taubner T., Keilmann F., Hillenbrand R. (2005). Nanoscale-Resolved
Subsurface Imaging
by Scattering-Type near-Field Optical Microscopy. Opt. Express.

[64] Voronin K. V., León I. H., Hillenbrand R., Nikitin A. Y. (2025). Quantitative Analytical
Spheroid Model for Scattering-Type Scanning Near-Field Optical Spectroscopy. Adv. Opt. Mater..

[65] Marsh D., Müller M., Schmitt F. J. (2000). Orientation of the Infrared Transition
Moments for an α-Helix. Biophys. J..

[66] Waeytens J., Mathurin J., Deniset-Besseau A., Arluison V., Bousset L., Rezaei H., Raussens V., Dazzi A. (2021). Probing Amyloid Fibril
Secondary Structures by Infrared Nanospectroscopy: Experimental and
Theoretical Considerations. Analyst.

[67] Intze A., Temperini M. E., Rupert J., Polito R., Veber A., Puskar L., Schade U., Ortolani M., Zacco E., Tartaglia G. G., Giliberti V. (2025). Effect of RNA on the Supramolecular
Architecture of α-Synuclein Fibrils. Biophys.
J..

