# Supplementary material for: Understanding In-Liquid Sample Environments for Infrared Nanospectroscopy of Soft Materials
Source: Anal Chem. 2026 Aug 31;98(36):26722–32. doi: 10.1021/acs.analchem.6c03284 (PMC13584630; doi:10.1021/acs.analchem.6c03284)
Supplement: Supplementary file 1 [file ac6c03284_si_001.pdf]

Supporting Information for:

Understanding In-liquid Sample Environments for Infrared Nano-spectroscopy of Soft Materials

Maria Eleonora Temperini[1,], Cecilia Spedalieri[2], Antonia Intze[3], Valeria Giliberti[3], Michele Ortolani[3], Janina Kneipp[2], Alexander Veber[1,2]*

[1] Institute for Electronic Structure Dynamics, Helmholtz-Zentrum Berlin für Materialien und Energie GmbH, Albert-Einstein-Straße 15, 12489 Berlin, Germany

[2] Department of Chemistry, Humboldt-Universität zu Berlin, Brook-Taylor-Straße 2, 12489 Berlin, Germany

[3] Department of Physics, Sapienza University of Rome, Piazzale Aldo Moro 5, 00185 Rome, Italy

Contents

S-I MODEL OF DIELECTRIC PERMITTIVITIES FOR SiN AND SiC 2
S-II PEAK FITTING AND BAND SHAPE ANALYSIS OF THE WATER BAND 4
S-III MODEL OF DIELECTRIC PERMITTIVITY FOR BSA, DNA MOLECULES AND α-SYNUCLEIN PROTEINS 6
S-IV COMPARISON OF ATR AND s-SNOM DNA SPECTRA 7
S-V CALCULATION OF THE PROBED MOLAR AMOUNT 8
S-VI SNOMPY MODEL 9

S-VII α-SYNUCLEIN NORMALIZED SPECTRA.. 10
S-VIII s-SNOM MODELING WITH DIFFERENT ELLIPSOID LENGTH 12
S-IX NEAR-FIELD DECAY CHARACTERIZATION BY RETRACT-CURVE MEASUREMENTS .. 13
S-X TECHINCAL DRAWING OF THE SAMPLE ENVIRONMENT ASSEMBLY FOR MEASUREMENTS IN LIQUID 15

S-I MODEL OF DIELECTRIC PERMITTIVITIES FOR SiN AND SiC

Figures S1a-b display the experimental reflectance spectrum of the 500 nm thick SiN membrane and SiC membrane (green curves), together with the corresponding fitting curves calculated for a thin film of thickness *d* (black curves). To extract the optical constants, the dielectric function $\varepsilon_{SiN}(\omega)$ of SiN was modeled as a combination of two Lorentzian oscillators and one causal Gaussian component consistent with the Kramers-Kronig relations:[1]

$$\varepsilon_{SiN}(\omega) = \varepsilon_{\infty} + \sum_{n=1}^{2} \varepsilon_{L,n}(\omega) + \varepsilon_G(\omega),$$

where ω is the light wavenumber and ε_{∞} is the high-frequency dielectric constant.

The Lorentzian contributions are defined as

$$\varepsilon_{L,n}(\omega) = \frac{S_n \Omega_n^2}{\Omega_n^2 - \omega^2 + i\gamma_n \omega},$$

with S_n representing the oscillator strength, Ω_n the resonance frequency and γ_n the damping factor.

While the imaginary part of the Gaussian component is given by

$$Im[\varepsilon_G(\omega)] = A(e^{-\frac{(\omega-\Omega_0)^2}{2\sigma^2}} - e^{-\frac{(\omega+\Omega_0)^2}{2\sigma^2}}),$$

with A representing the peak amplitude, Ω_0 the resonance frequency and σ the Gaussian width.

The corresponding real part is obtained through the Kramers-Kronig transformation. The optimization yielded an effective thickness *d* = 480 nm (consistent with the nominal value of 500 nm) and the following parameters: ε_{∞} =4.76, S_1=1.60, Ω_1=902.00 cm^{-1}, γ_1=153.00 cm^{-1}, S_2=1.14,

Ω_2=468.00 cm^{-1}, γ_2=158.00 cm^{-1}, A=9.07, Ω_0=822.00 cm^{-1} and σ=95.20 cm^{-1}. We found that the Gaussian component improves the fit quality and can be explained by the amorphous nature of the SiN used for membrane fabrication.

For the SiC membrane, a single Lorentzian oscillator model was employed:

$$\varepsilon_{SiC}(\omega) = \varepsilon_\infty + \frac{S\Omega^2}{\Omega^2 - \omega^2 + i\gamma\omega}$$

with resulting parameters of ε_∞=6.50, S=3.12, Ω=795.50 cm^{-1}, γ=6.93 cm^{-1}. The real and imaginary parts of the retrieved dielectric functions for both materials are plotted in Figure S1c-d.

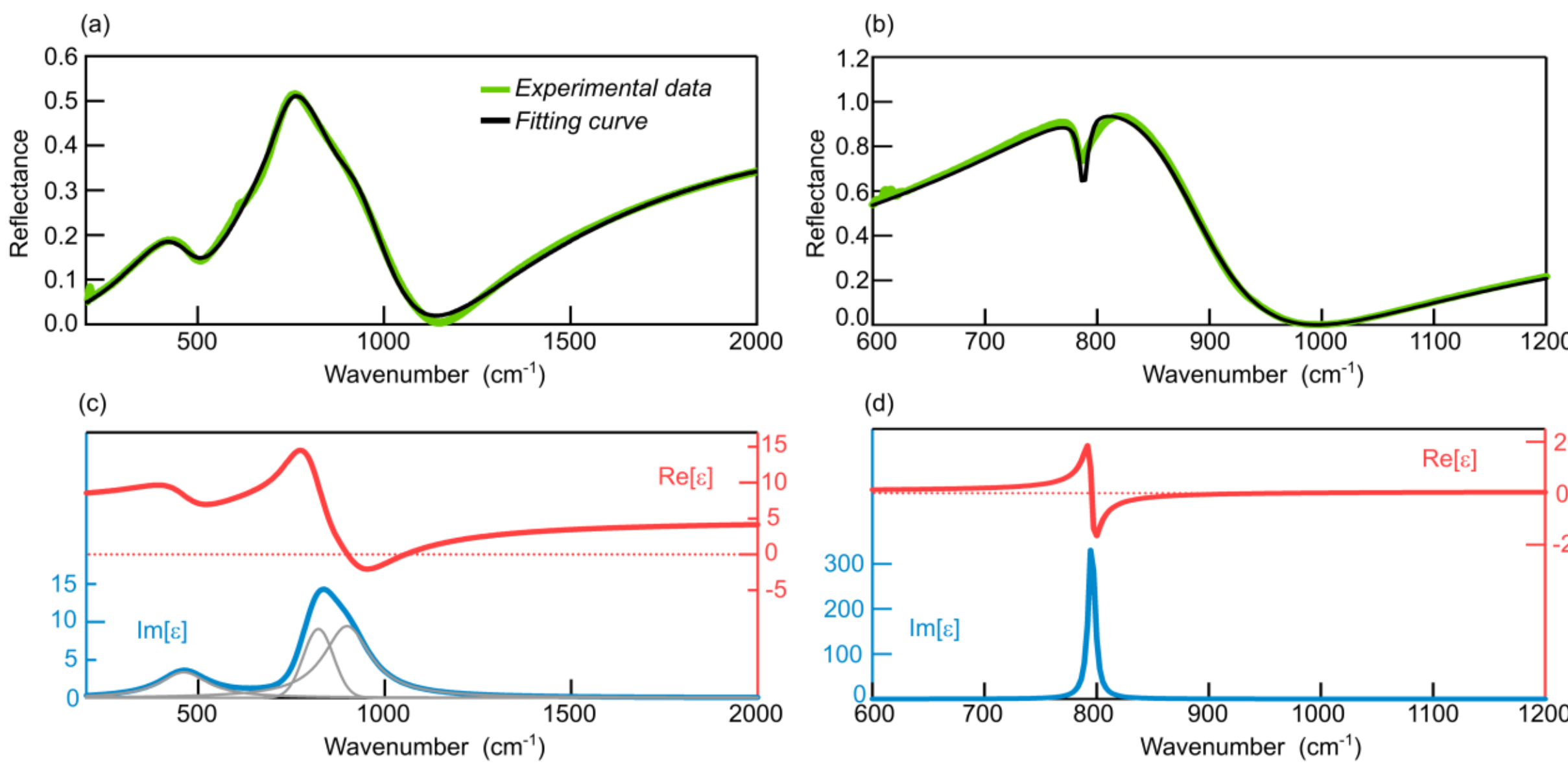

Figure S1. FTIR reflectance spectra (green curves) and corresponding model fits (black curves) for the SiN (a) and SiC membranes(b), respectively. Corresponding real (red curve, right axis) and imaginary (light blue curve, left axis) parts of the dielectric functions obtained from the model for SiN (c) and SiC (d) materials, respectively. For the imaginary part of SiN dielectric function, the single components are also shown (gray curves).

S-II PEAK FITTING AND BAND SHAPE ANALYSIS OF THE WATER BAND

To evaluate the peak position and width of the OH bending vibrational band of water, the experimental spectra were fitted using a Gaussian profile. From the fitted curves (gray curves in Figure 2f of the main text), the peak position ω_0 and the full width at half maximum (FWHM) were extracted.

To assess possible deviations from a symmetric Gaussian line shape, the spectral positions corresponding to half of the maximum intensity were also determined directly from the experimental spectra on the low- (ω_L) and high-frequency (ω_R) sides of the peak. The symmetry of the band was evaluated by comparing the distances of these points from the peak center, $\Delta L = \omega_0 - \omega_L$ and $\Delta R = \omega_R - \omega_0$. Differences between these two values provide a quantitative estimate of the peak asymmetry, resulting in a minor asymmetry ($\Delta L - \Delta R = 6\ cm^{-1}$) of the peak for the SiN membrane. The parameters obtained from the near-field measurements of the phase-shift on SiN and SiC membranes were also compared with those extracted from the far-field ATR absorbance spectra. The resulting values are reported in Table S1. To evaluate the reproducibility of the OH bending band linewidth, independent measurements were fitted and analyzed (12 measurements for SiN and 9 measurements for SiC). The resulting average FWHM values are $83.2 \pm 12.6\ cm^{-1}$ for SiN and $97.8 \pm 12.4\ cm^{-1}$ for SiC. This additional analysis confirms that the linewidth difference is not associated with a single measurement, although some variability is observed among independent acquisitions.

	ω_0	FWHM	ΔL	ΔR
**s-SNOM with 10 nm SiN**	1646.6	65.9	37	31
**s-SNOM with 30 nm SiC**	1642.4	94.7	47	47
**ATR**	1643.3	88.8	42	44

Table S1. Comparison of the parameters ω_0, FWHM, ΔL and ΔR (cm^{-1}) for the water band extracted from Gaussian fits and experimental spectra in s-SNOM measurements on SiN and SiC membranes and in far-field ATR data.

The analysis was performed on the s-SNOM phase-shift signal. It is known that, for weak oscillators, the best agreement is typically observed between the near-field nano-absorption spectra, calculated as $a_2 = s_2 * \sin(\phi_2)$, and the far-field absorbance spectra. In our case, the nano-absorption spectra calculated from the experimental data show only minor differences with respect to the phase-shift spectra (Figure S2), consistent with previous observations for thin organic films.[3] Therefore, both the phase-shift and the nano-absorption spectra can be equivalently used for comparison.

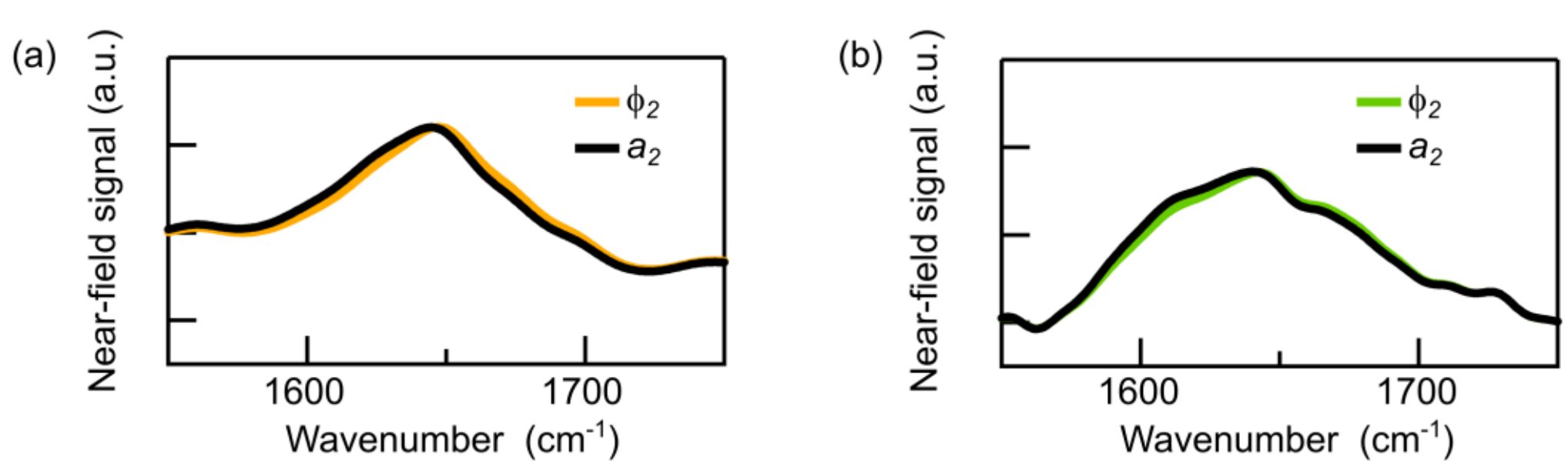

Figure S2. (a) and (b) Comparison of the near-field nano-absorbance a_2 and phase-shift ϕ_2 signal for the water band peak measured through SiN and SiC, respectively. The data are normalized to the peak maximum.

S-III MODEL OF DIELECTRIC PERMITTIVITY FOR BSA, DNA MOLECULES AND α-SYNUCLEIN PROTEINS

Figures S3a-b show the ATR-FTIR spectra of BSA and DNA solutions, referenced using the pure water spectrum. To derive the complex dielectric functions ε(ω) for both biomolecules, the experimental data were fitted using a thin-film transmission model. The dielectric response (Fig. S3c-d) was reconstructed by employing a multi-oscillator approach: 26 Lorentzian oscillators were used to model the complex spectral features of DNA, while 13 oscillators were sufficient for the BSA response.

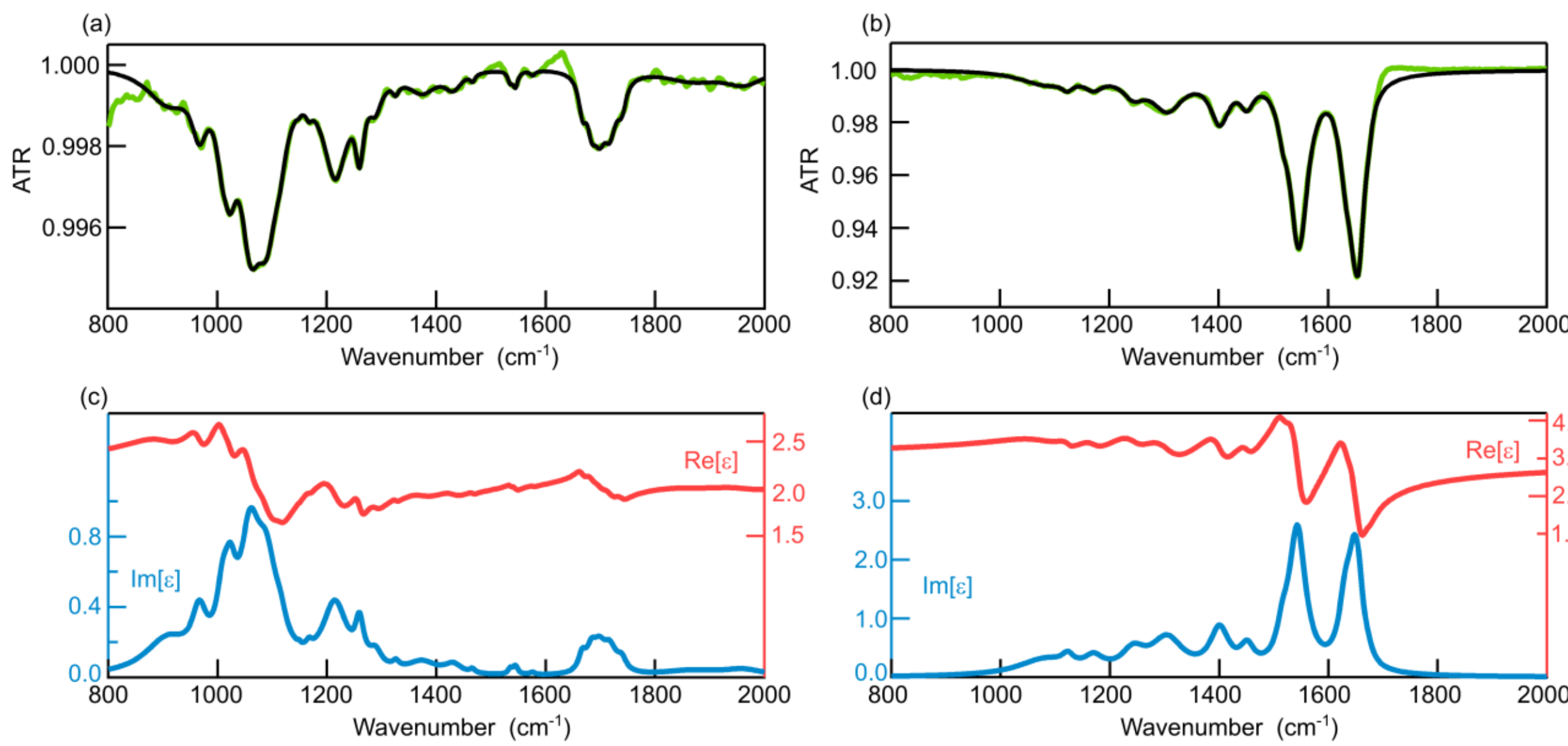

Figure S3. (a) and (b) ATR-FTIR spectra (green curves) and corresponding model fits (black curves) for the DNA and BSA solutions, respectively. (c) and (d) Corresponding real (red curve, right axis) and imaginary (light blue curve, left axis) parts of the dielectric functions obtained from the model for DNA and BSA, respectively.

A minor discrepancy between the experimental data and the model is observed in the 1600-1700 cm^{-1} region. This divergence can be attributed to experimental artifacts arising from the referencing process. In this spectral range, the high absorbance of the OH bending mode of water

makes the reference subtraction highly sensitive to even minimal variations, often leading to residual features in the normalized spectra. Despite these localized artifacts, the model provides a reliable representation of the biomolecular dielectric functions.

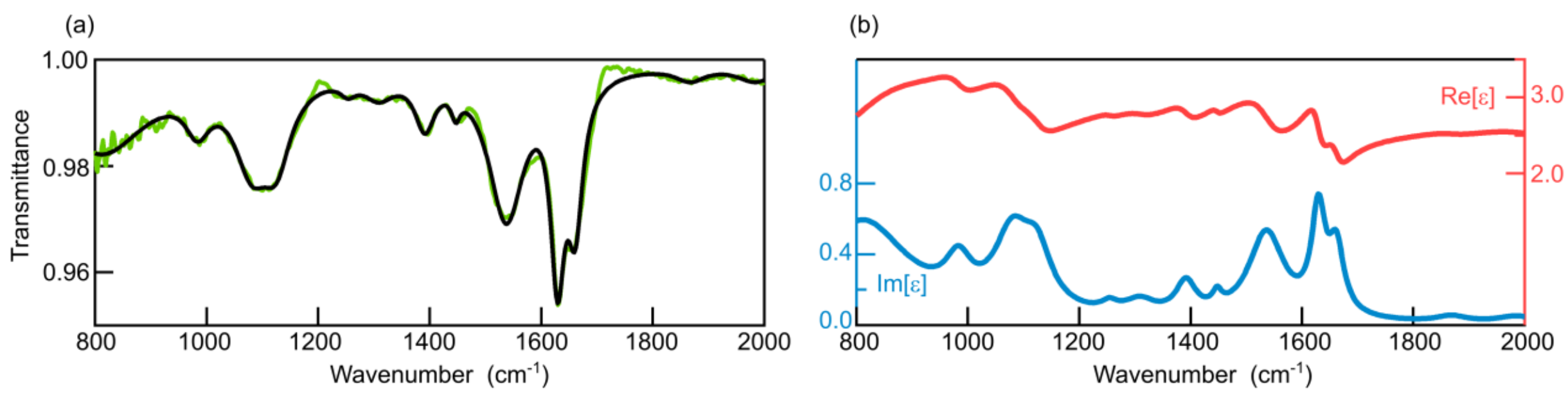

Figure S4. (a) FTIR transmittance spectrum (green curve) and corresponding model fit (black curve) for α-synuclein fibrils. (b) Corresponding real (red curve, right axis) and imaginary (light blue curve, left axis) part of the dielectric functions obtained from the model.

Figure S4a shows the transmission spectrum (green curve) of α-synuclein fibrils deposited on a calcium fluoride (CaF_2) window and dried, referenced against a clean CaF_2 substrate. The black curve represents the fit obtained by modeling the trasmittance of a 100 nm thick film of α-synuclein, reconstructed using a sum of 13 Lorentzian oscillators. The resulting dielectric function is reported in Figure S4b.

S-IV COMPARISON OF ATR AND s-SNOM DNA SPECTRA

To further investigate the origin and reproducibility of the weak spectral features observed in the s-SNOM spectra of DNA acquired through the SiN and SiC membranes (Figures 4b and 4d of the main text), the near-field spectra were compared with far-field ATR absorbance measurements. Figure S5 reports the comparison between the normalized s-SNOM phase-shift signal and the ATR absorbance spectrum of DNA. The two main vibrational bands at approximately 1083 cm^{-1} and 1230 cm^{-1}, assigned to the PO_2^- symmetric and asymmetric stretching modes, respectively, are clearly observed in the s-SNOM spectra acquired through the SiC membrane. Their identification

is instead hampered in the SiN measurements due to the overlap with the SiN resonances in the same spectral range. In addition, several weaker spectral features are detected in the s-SNOM response at higher frequencies. For the SiC membrane, many of these features occur at frequencies corresponding to vibrational bands present in the ATR spectrum (marked in Figure S5), supporting their assignment to DNA vibrational modes rather than experimental artifacts. For the SiN membrane, the correspondence with the ATR spectrum is less evident.

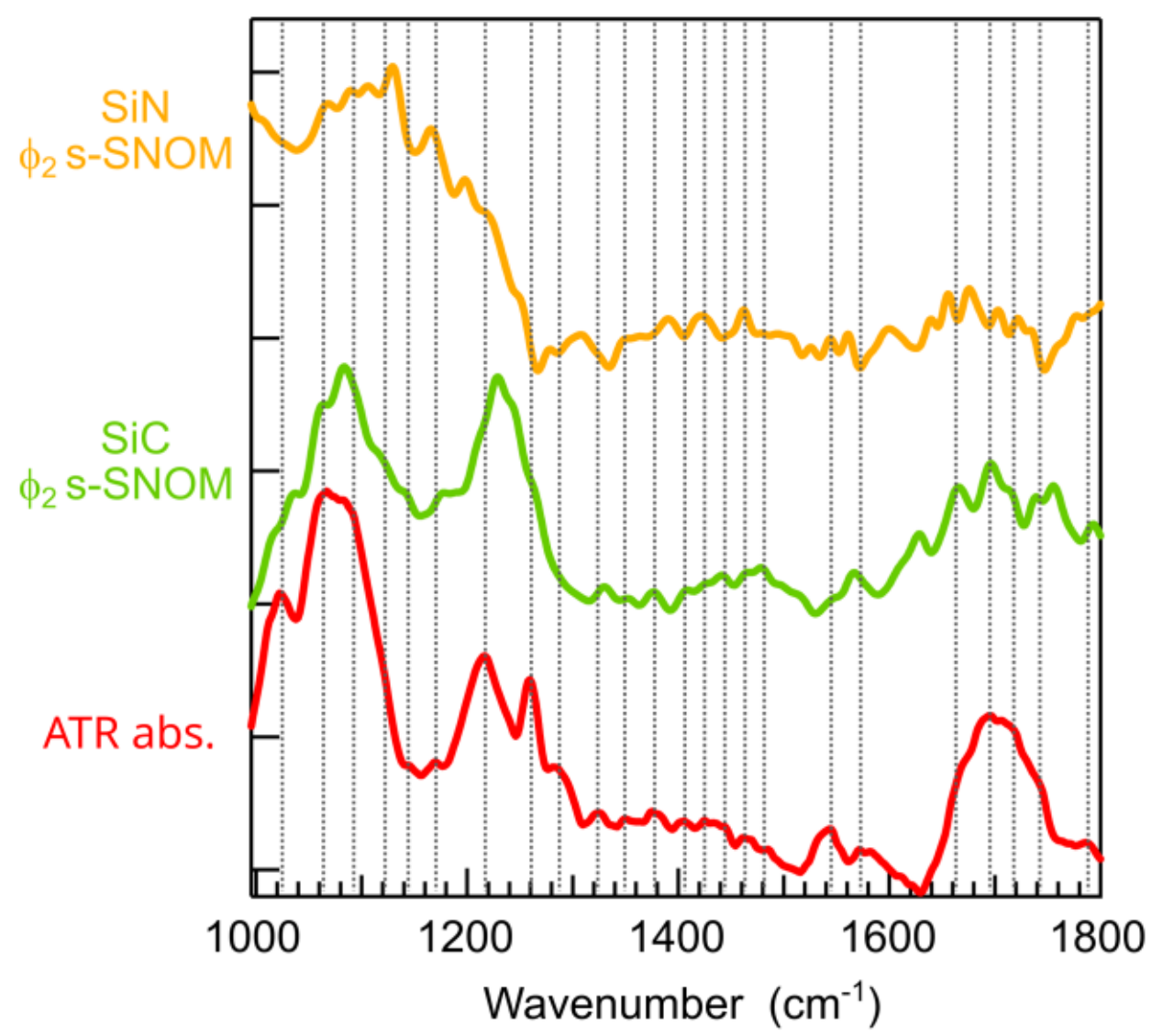

Figure S5. Normalized s-SNOM phase-shift signal (φ_2) of DNA acquired through the SiN (yellow) and SiC (green) membrane compared with the corresponding ATR spectrum (red). The spectral features associated with DNA vibrational modes are highlighted.

S-V CALCULATION OF THE PROBED MOLAR AMOUNT

In s-SNOM, the near-field interaction is localized within a nanoscale volume whose characteristic dimensions are determined by the tip geometry and the tip-sample coupling. For estimating the number of molecules contributing to the signal, we approximate the effective sensing volume using the characteristic interaction length extracted from retract curve measurements. For the 2nd harmonic signal, the 1/5 decay criterion corresponds to an interaction length *d* of approximately 85 nm (see Section S-IX). Since the near-field signal decays

continuously with increasing tip-sample distance, this value should be regarded as a conservative approximation of the spatial extent of the interaction rather than a sharply defined probing depth. The interaction region can be approximated as a cylindrical volume $V=\pi r^2 d$ with lateral dimensions r defined by the tip apex. In our membrane configuration, the presence of a dielectric layer above the solution further reduces the portion of the near-field that reaches the liquid phase. For the 10 nm SiN membrane, the probing depth within the solution is estimated to be~ 75 nm, yielding an interaction volume of approximately 9.0×10^{-16} mL. For the thicker 30 nm SiC membrane, a smaller fraction of the near-field penetrates into the solution, corresponding to a probing depth of~ 55 nm and an interaction volume of about 5.5×10^{-16} mL. Using a BSA concentration of 0.55 g/mL (molecular weight 66.5 kDa), these volumes correspond to approximately 7.5 zmol of protein for the SiN membrane and 5.5 zmol for the SiC membrane. For DNA solutions (10 mg/mL) measured through the SiC membrane and assuming a molecular weight range of 50-100 kDa, the amount of molecules within the probed volume is estimated to be on the order of $5.5\text{-}13.0 \times 10^{-2}$ zmol.

S-VI SNOMPY MODEL

To support the experimental findings, numerical simulations were performed using the Finite Dipole Model (FDM) with the charge-average method[5] to determine the effective tip-sample polarizability α_{eff}. This parameter describes the complex near-field coupling between the probe and the multilayered sample system. The complex scattering coefficient σ was calculated as:

$$\sigma = \frac{E_{scatt}}{E_{in}} = (1 + c_r r_p)^2 \, \alpha_{eff}$$

In this expression, r_p is the far-field Fresnel reflection coefficient for p-polarized light. The empirical factor c_r describes the detected strength of the reflected light relative to the incident light. This factor depends on the specific microscope setup, including optical alignment and collection

efficiency. In our modeling, c_r was adjusted to match the experimental signal amplitude when referencing the sample response to the chosen material (Si or water). This calibration step is necessary to compensate for variations in the detected far-field contribution that may occur throughout the experimental measurements, ensuring that the simulated contrast and absolute amplitude levels accurately reflect the measured data. Regarding the references, a constant dielectric function of 11.7 was used for Si, given its flat spectral response in the mid-IR region. A dispersive dielectric function was instead employed for SiO_2 and water to account for the absorption bands and the frequency-dependent refractive index across the investigated spectral range.[6,7]

S-VII α-SYNUCLEIN NORMALIZED SPECTRA

We report here in Figure S6 the experimental spectra shown in Figure 5c and the corresponding modeled spectra shown in Figure 5d of the main text, acquired on the α-synuclein sample under the SiN membrane. The spectra were normalized to the intensity of the broad band around 1645 cm^{-1}, corresponding to the overlapping water bending and amide I region, to facilitate the comparison of the spectral lineshape and the relative contribution of the different spectral components between spot 1 and spot 2 (Figure S6a). As shown in Figure S6b, the spectrum acquired at spot 2 (red curve) exhibits a larger relative contribution from water compared to spot 1 (yellow curve), as indicated by the shape and width of the band around 1645 cm^{-1}, which closely resembles the OH bending mode of pure water (light blue curve). Conversely, the spectrum acquired at spot 1 displays a narrower band in the amide I region and a more pronounced contribution from the amide II band around 1565 cm^{-1}, consistent with a larger relative protein contribution. The same trend is reproduced in the normalized modeled spectra shown in Figure S5c. In the model, the signal from the bright spot 1 is represented by an effective α-synuclein fibril layer with a thickness of 50 nm, whereas the dark spot 2 is initially modeled as an effective 30 nm

protein layer separated from the SiN membrane by a 30 nm water layer. However, the water/protein configuration capable of reproducing the observed spectral response is not unique. As shown in Figure S5d, an alternative configuration consisting of a thinner 10 nm protein layer located directly beneath the SiN membrane provides a comparable spectral response to the configuration with a 30 nm water gap and a 30 nm protein layer. These results indicate that the model supports the interpretation that the spectral variations observed between regions with different optical contrast originate from a different relative contribution of water and protein within the near-field probed volume, while the exact geometrical distribution of the protein beneath the membrane cannot be uniquely determined from the spectra alone.

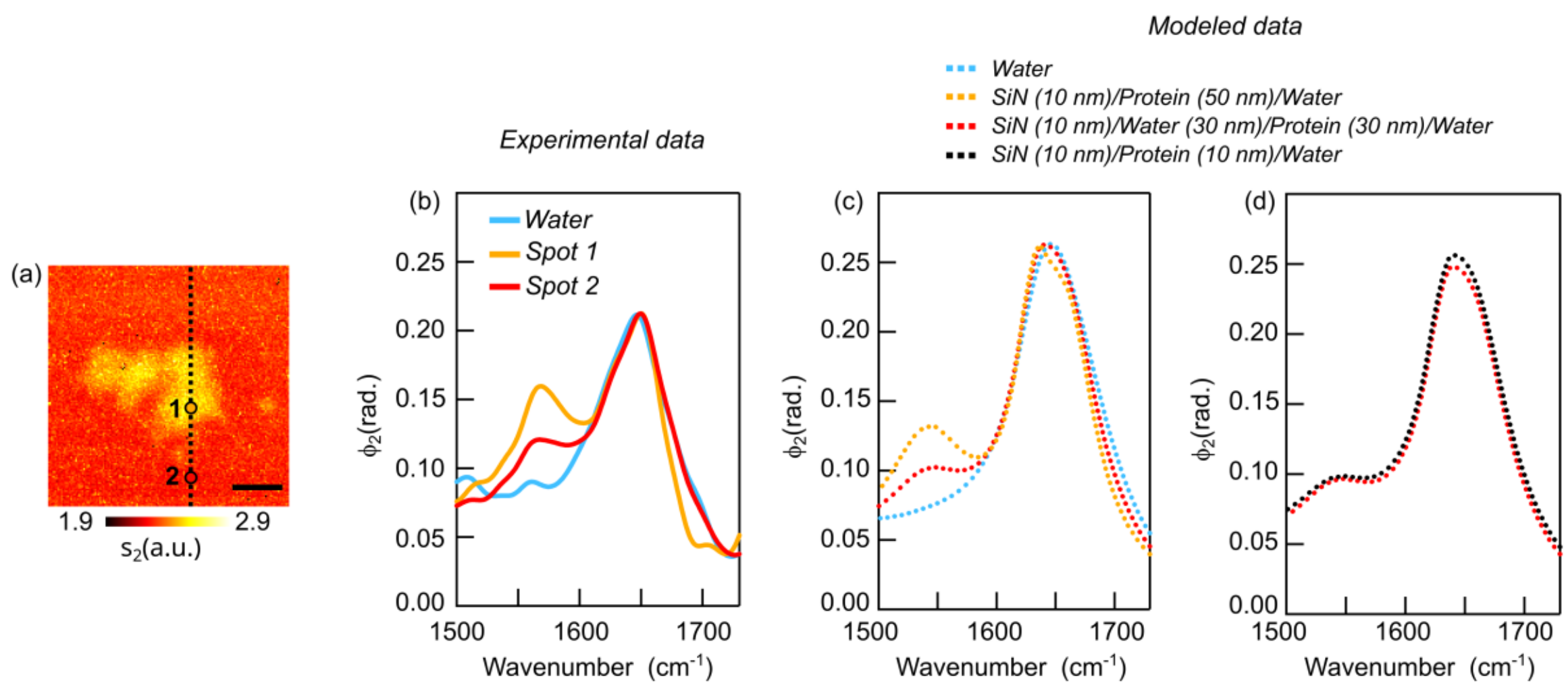

Figure S6. (a) s-SNOM optical amplitude s_2 map of α-synuclein fibrils in aqueous solution acquired through a SiN membrane. Scale bar: 1 µm. (b) Near-field phase φ_2 spectra acquired at the locations marked as spot 1 and spot 2 in (a). The light blue curve corresponds to the pure water reference spectrum. The spectra are normalized to the intensity of the band around 1645 cm^{-1}. (c) Modeled φ_2 spectra corresponding to the configurations used in the main text for spot 1 (yellow) and spot 2 (red), together with the simulated spectrum of pure water (light blue). The spectra are normalized to the intensity of the band around 1645 cm^{-1}. (d) Comparison of modeled φ_2 spectra

for two alternative protein/water configurations used to describe spot 2: a 30 nm protein layer separated from the membrane by a 30 nm water layer (red) and a 10 nm protein layer directly beneath the SiN membrane (black).

S-VIII s-SNOM MODELING WITH DIFFERENT ELLIPSOID LENGTH

To evaluate the influence of the tip geometry on the simulated near-field spectra, additional FDM calculations were performed by varying the effective ellipsoid length *L*, used to approximate the elongated geometry of the AFM tip. The effective ellipsoid length was varied while keeping all other model parameters unchanged, including the tip radius (60 nm) and tapping amplitude (115 nm), which were determined from the experimental conditions. Figure S7 shows the simulated spectra obtained for different ellipsoid lengths in the membrane/water configuration.

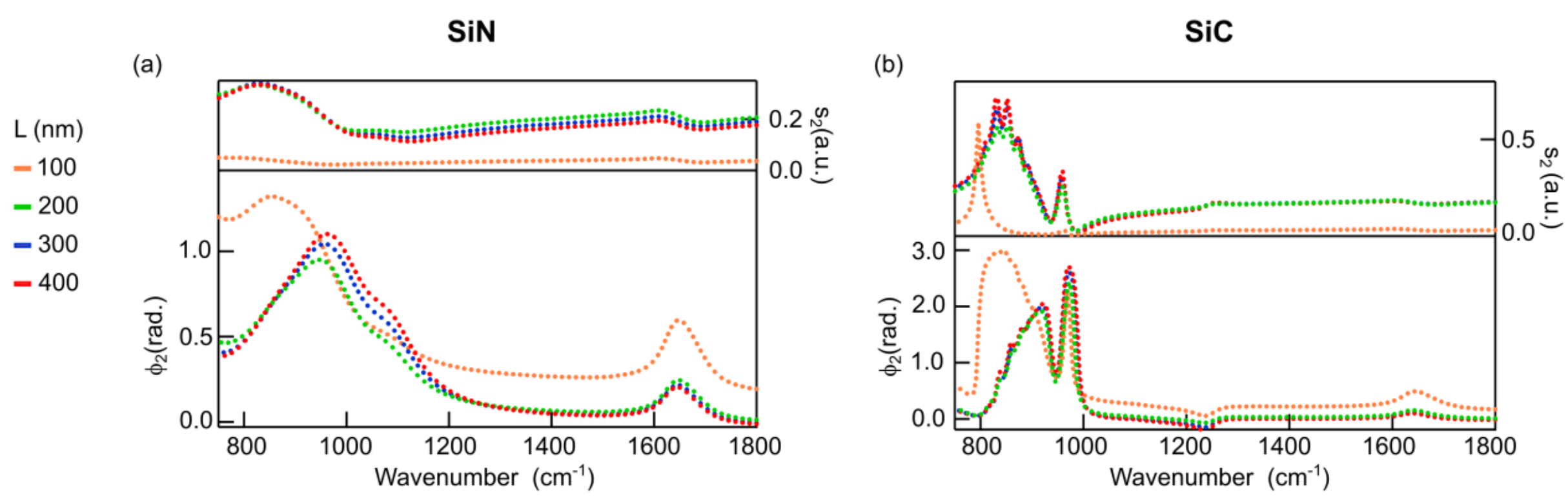

Figure S7. (a) and (b) modeled s-SNOM signal of water under SiN and SiC membranes, respectively, obtained varying the ellipsoid tip length *L*. The top panels show the simulated amplitude signal s_2 (right axes) and the bottom panels show the corresponding phase signal φ_2 (right axes).

Variations in the ellipsoid length affect both the near-field signal intensity and the relative contribution of the different spectral components. In particular, shorter ellipsoid lengths improve the agreement with the membrane resonances in the case of SiC, whereas longer ellipsoid lengths provide a better agreement of the water band intensity in the phase signal. The values reported in

the main text were selected to provide primarily with the best overall agreement with the experimental spectra for water and molecular absorption features and were kept fixed for all datasets presented in the work.

S-IX NEAR-FIELD DECAY CHARACTERIZATION BY RETRACT-CURVE MEASUREMENTS

To verify the near-field origin of the detected signal, retract curve measurements were performed using the 2nd, 3rd and 4th demodulation harmonics. Figure S8a shows the normalized retract curves measured on SiC membrane with water underneath, where the signal is normalized to its value at tip-sample contact. As expected, the higher demodulation harmonics exhibit a steeper decay with increasing tip-sample distance z and a progressively stronger suppression of the far-field contribution. The asymptotic value reached at large distances is reported in Figure S8a and was used to estimate the residual far-field background for each harmonic. After subtraction of this background, the normalized near-field decay curves are shown in Figure S8b. From these curves, the characteristic interaction lengths were estimated using both the 1/e and the 1/5 criterion, corresponding to the distances at which the near-field signal decreases to 37% and 20% of its maximum value, respectively. While the 1/e value represents the characteristic exponential decay length, the 1/5 value provides a more conservative estimate of the spatial extent over which the near-field interaction remains significant. Beyond the analysis of the retract curves, the spectroscopic performance of the different demodulation harmonics was assessed by comparing spectra acquired through the SiC membrane. Figure S9 compares the BSA spectra acquired through the SiC membrane using 2nd, 3rd and 4th harmonic demodulation signals. The characteristic amide I and amide II vibrational bands are still visible in the 3rd harmonic, confirming that the measured spectral response originates from the near-field interaction. At the same time, as expected, the signal-to-noise ratio decreases for higher harmonics because of the reduced

demodulated signal intensity. For this reason, the 2nd harmonic was adopted throughout this work, since it provides a higher signal quality while still efficiently suppressing the far-field background.

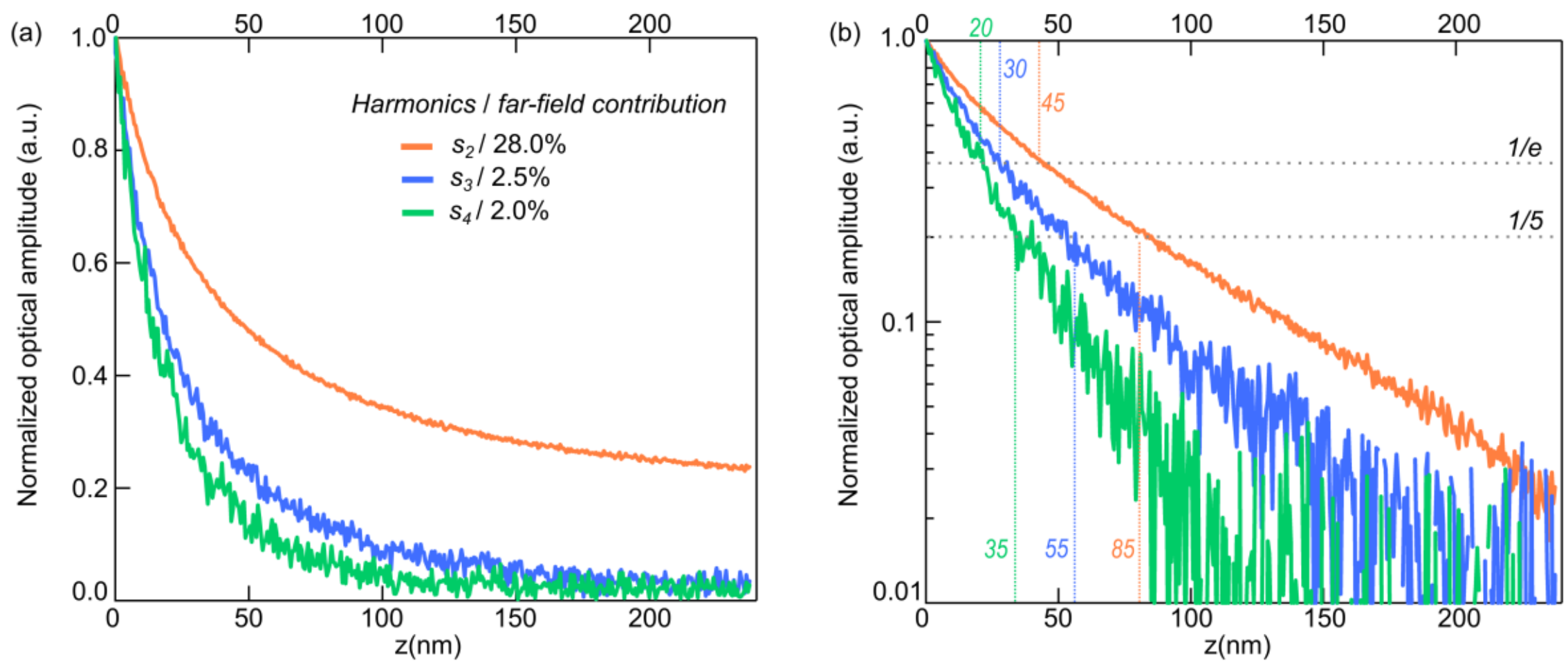

Figure S8. (a) Normalized retract curves acquired on a SiC membrane for the 2nd (orange), 3rd (blue) and 4th (green) demodulation harmonics. (b) Background-corrected retract curves after subtraction of the asymptotic far-field contribution. Dashed horizontal lines indicate the 1/e (37%) and 1/5 (20%) signal levels used to estimate the characteristic interaction lengths.

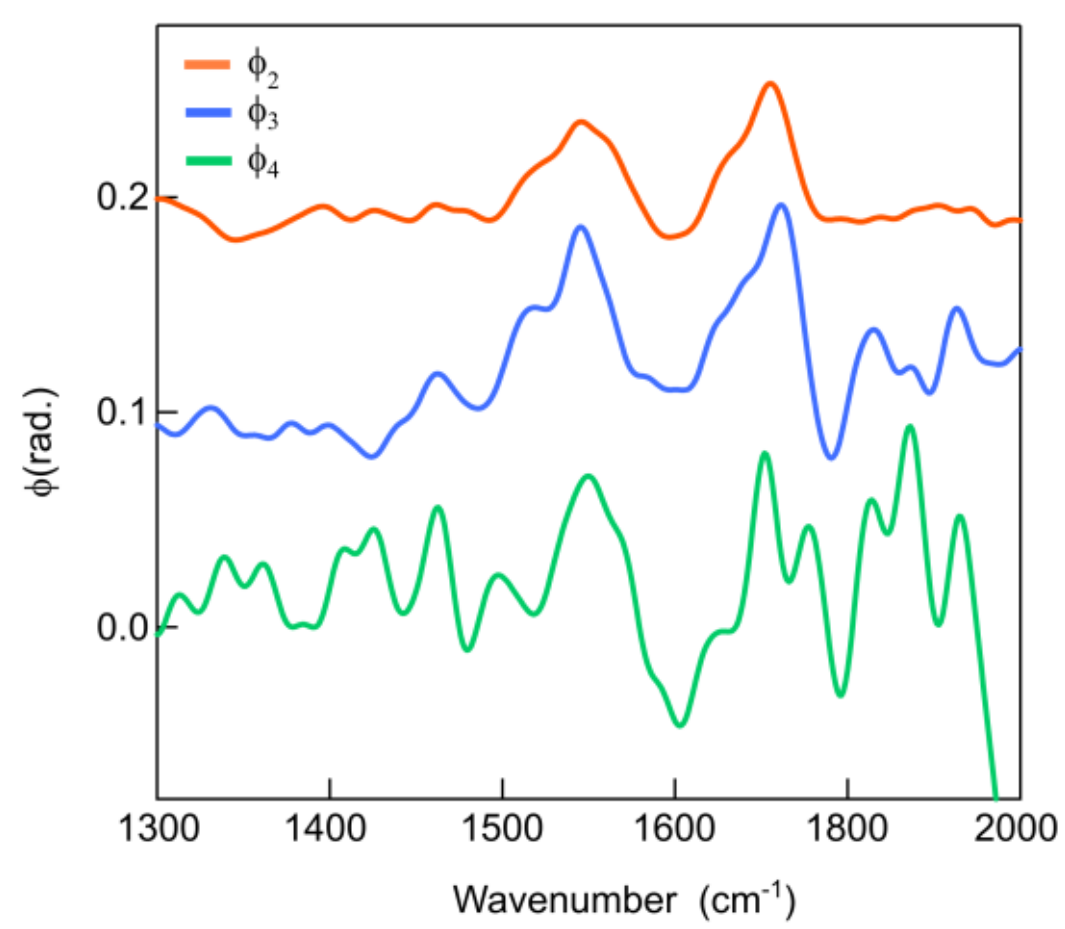

Figure S9. s-SNOM phase spectra of BSA measured through the SiC membrane using the 2nd (orange), 3rd (blue) and 4th (green) demodulation harmonics. The curves are vertically offset by equal increments of 0.1 radians for clarity

S-X TECHINCAL DRAWING OF THE SAMPLE ENVIRONMENT ASSEMBLY FOR MEASUREMENTS IN LIQUID

Figure S10 shows the technical drawing of the custom-made aluminum holder used for the liquid measurements. The membrane frame is positioned and sealed over the central through-hole, thereby forming the top wall of the liquid chamber. On the back side of the holder, a secondary filling port is connected to the central opening through a narrow channel, allowing the liquid solution to be introduced into the chamber while minimizing the mechanical stress on the suspended membrane. After the solution is introduced, the back-side filling port is sealed with adhesive tape to prevent leakage during the measurements.

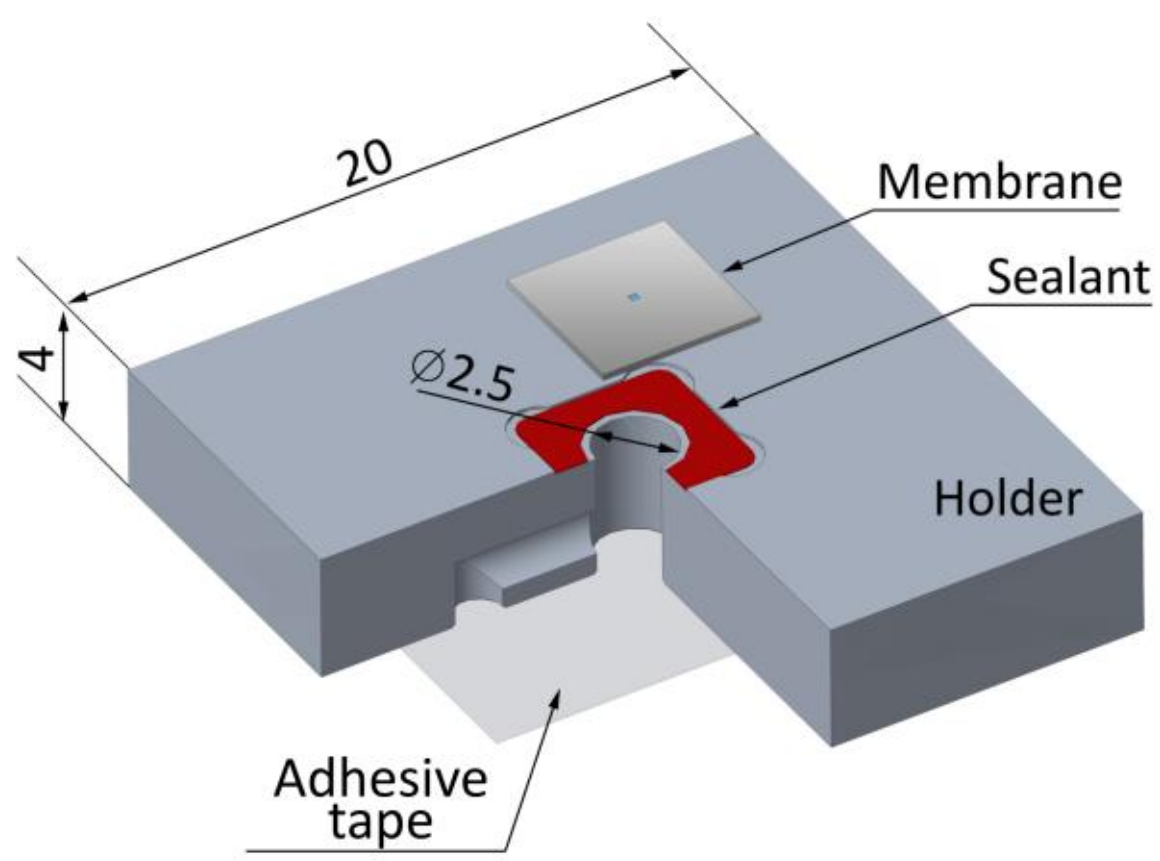

Figure S10. Exploded-view drawing of the environment assembly for measurements in liquid. The membrane frame is placed on top of the central hole of the aluminum holder after application of the sealant (red film). All dimensions are reported in millimeters.

REFERENCES

(1) Keefe, C. D. Curve fitting Imaginary Components of Optical Properties: Restrictions on the Lineshape Due to Causality. *J. Mol. Spectrosc.* **2001**, *205* (2), 261–268. https://doi.org/10.1006/jmsp.2000.8254.

(2) *Speqqle*. https://www.speqqle.com/.

(3) Mastel, S.; Govyadinov, A. A.; De Oliveira, T. V. A. G.; Amenabar, I.; Hillenbrand, R.

Nanoscale-Resolved Chemical Identification of Thin Organic Films Using Infrared near-Field Spectroscopy and Standard Fourier Transform Infrared References. *Appl. Phys. Lett.* **2015**, *106* (2). https://doi.org/10.1063/1.4905507.

(4) Taubner, T.; Keilmann, F.; Hillenbrand, R. Effect of Tip Modulation on Image Contrast in Scattering-Type near-Field Optical Microscopy. *J. Korean Phys. Soc.* **2005**, *47* (SUPPL. 1), 213–216.

(5) Vincent, T.; Liu, X.; Johnson, D.; Mester, L.; Huang, N.; Kazakova, O.; Hillenbrand, R.; Boland, J. L. Snompy: A Package for Modelling Scattering-Type Scanning Near-Field Optical Microscopy. **2024**.

(6) Hale, G. M.; Querry, M. R. Optical Constants of Water in the 200-Nm to 200-Mm Wavelength Region. *Appl. Opt.* **1973**, *12* (3), 555. https://doi.org/10.1364/ao.12.000555.

(7) Franta, D.; Nečas, D.; Ohlídal, I.; Giglia, A. Optical Characterization of SiO 2 Thin Films Using Universal Dispersion Model over Wide Spectral Range . *Opt. Micro- Nanometrology VI* **2016**, *9890*, 989014. https://doi.org/10.1117/12.2227580.
